# Animal residues found on tiny Lower Paleolithic tools reveal their use in butchery

**DOI:** 10.1038/s41598-019-49650-8

**Published:** 2019-09-10

**Authors:** Flavia Venditti, Emanuela Cristiani, Stella Nunziante-Cesaro, Aviad Agam, Cristina Lemorini, Ran Barkai

**Affiliations:** 1 Institute of Archaeology, Tel Aviv University, Tel Aviv, 69978 Israel; 2 DANTE Diet and Ancient Technology Laboratory, Sapienza, University of Rome, Via Caserta 6, Rome, 00161 Italy; 3 Scientific Methodologies Applied to Cultural Heritage (SMATCH), ISMN-CNR c\o Dept. of Chemistry, Sapienza, University of Rome, P.le Aldo Moro, Rome, 00185 Italy; 4 Department of Classics, LTFAPA Laboratory, Sapienza, University of Rome, P.le Aldo Moro, Rome, 00185 Italy

**Keywords:** Archaeology, Archaeology

## Abstract

Stone tools provide a unique window into the mode of adaptation and cognitive abilities of Lower Paleolithic early humans. The persistently produced large cutting tools (bifaces/handaxes) have long been an appealing focus of research in the reconstruction of Lower Paleolithic survival strategies, at the expenses of the small flake tools considered by-products of the stone production process rather than desired end products. Here, we use use-wear, residues and technological analyses to show direct and very early evidence of the deliberate production and use of small flakes for targeted stages of the prey butchery process at the late Lower Paleolithic Acheulian site of Revadim, Israel. We highlight the significant role of small flakes in Lower Paleolithic adaptation alongside the canonical large handaxes. Our results demonstrate the technological and cognitive flexibility of early human groups in the Levant and beyond at the threshold of the departure from Lower Paleolithic lifeways.

## Introduction

In the Levant, the Acheulian cultural complex persisted for over one million years (ca 1,400,000 to 400,000 years ago) and is the main human mode of adaptation of the Lower Paleolithic period^1^, a long and successful epoch of fundamental transformations in human behavioral and biological evolution^2,3^. The Acheulian is often associated with the production and use of bifaces or large cutting tools (LCTs, e.g., handaxes and cleavers), considered the hallmark of their time^4^. The persistence of LCTs through time and space has been interpreted by some scholars as a technological stasis reflecting lack of creativity^5^. However, the discovery of flakes and flake tools smaller than 2–3 cm at Acheulian sites gives us reason to reconsider Lower Paleolithic lithic traditions and variability^6–9^. We show how these tiny tools, discovered at the Revadim site alongside larger flakes and large cutting tools (for details see supplementary materials), are part of a varied and pre-planned tool-kit produced for specific stages of the animal butchery process. As such, these tools are part of a set of early human adaptations that included other cognitively complex behaviors such as the use of fire and big-game hunting. Acheulian lithic variability itself is characterized by varied use of stone types, the use of hard and soft hammers, the application of predetermined flake production technologies, and the production of tiny sharp flakes by recycling older items^4,10,11^.

The discovery of these varied tool-kits consisting of large and small flakes^6,12^ (while handaxes were not always present^6,13^) has led to recent attempts to technologically reconstruct small flake production to better understand the role of these tiny tools^6–9^. In this context, the late Lower Paleolithic Acheulian site of Revadim provides an unprecedented opportunity to explore for the first time the production and use of small flake tool-kits produced by means of lithic recycling^14^ (for details see Supplementary materials). In addition to the plethora of stone artefacts, thousands of butchered animal bones were also retrieved from the site, including those of elephants, who played a prominent role in human diet and whose bones served as raw material for tools^15^. The site also yielded direct evidence for the use of large stone tools in animal processing^16^.

**Figure 1:**
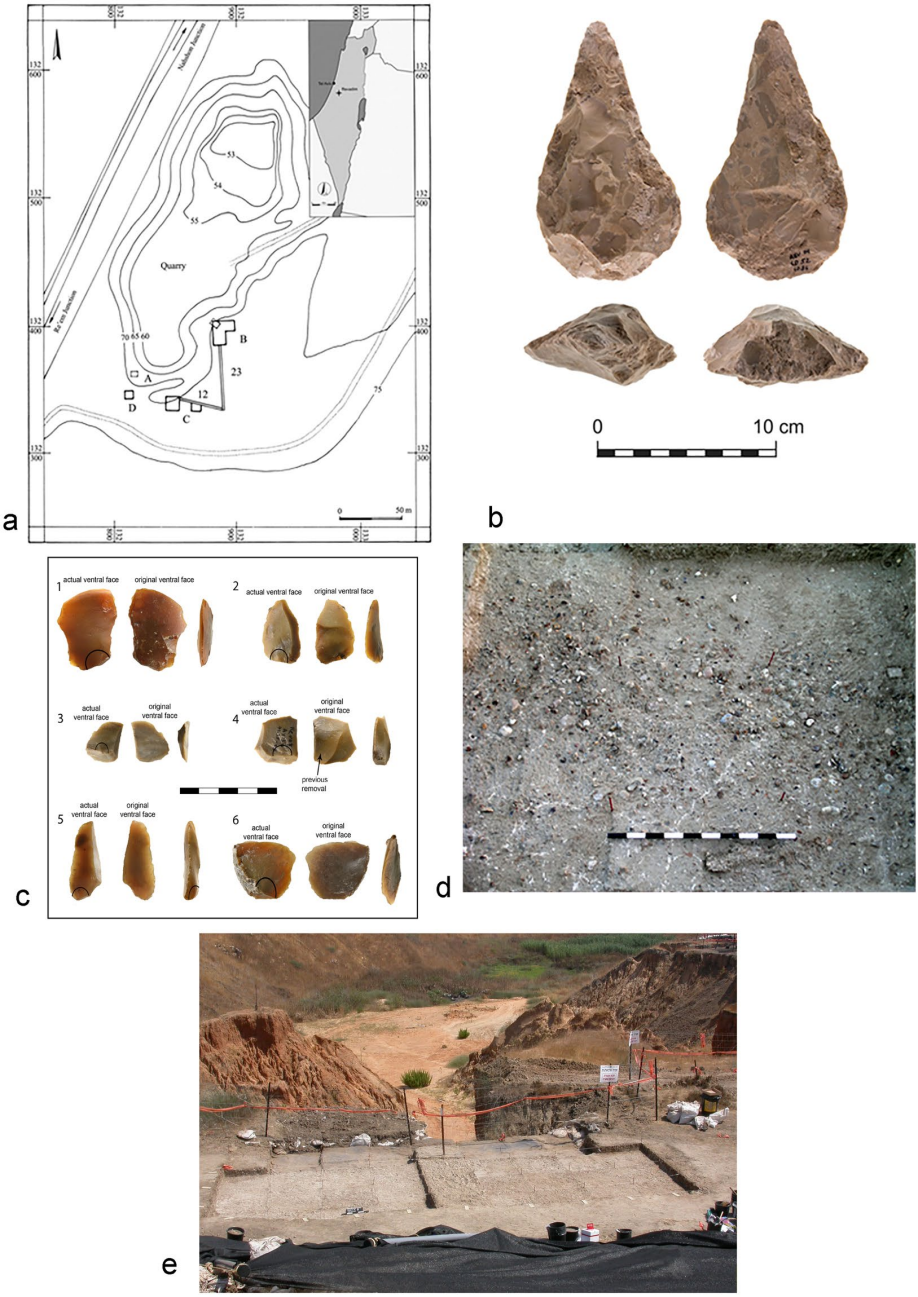
Geographical and archaeological settings of the Revadim site. (**a**) The location and excavation areas of the late Acheulian site of Revadim^14^. (**b**) Handaxe from the layer C3 assemblage^14^. (**c**) A group of small flakes produced by lithic recycling^14^. (**d**) Close-up of layer C3. (**e**) Area C, view to the north east.

Revadim is an open-air site dated to ca 500–300 kyr^17^ (Fig. 1a,e and Supplementary materials). Its attribution to the Late Acheulian cultural complex of the Levant is based on the stone tool assemblages, the presence of straight-tusked elephant bones, and preliminary radiometric dates. Most of the small flakes were excavated from archaeological layer 3 in Area C^14^ (Fig. 1d). The density of stone artefacts and animal bones unearthed at this stratum is probably the result of a number of discrete occupation events accumulated to a palimpsest.

**Figure 2:**
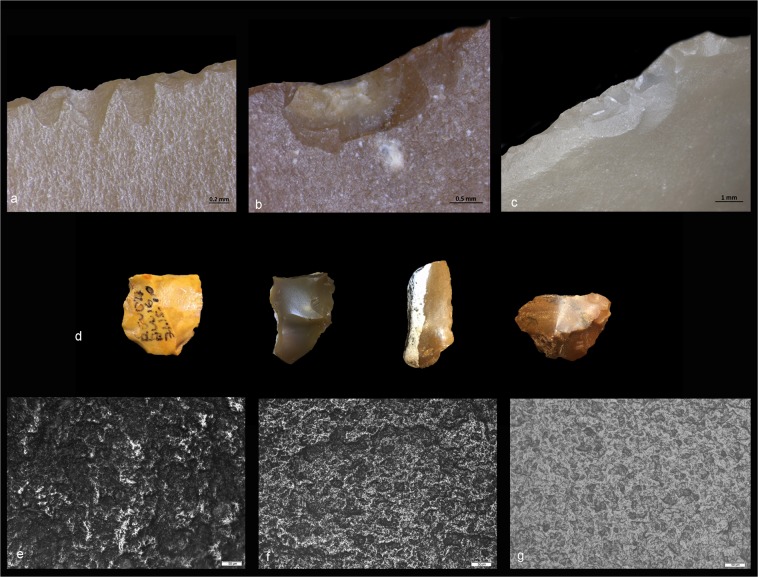
Different types of alterations observed on the products of recycling. (**a**) Post-depositional and (**b**,**c**) post-excavation edge scarring. (**d**) Four small flakes from Revadim exhibiting evidence of color change due to patination. (**e**–**g**) OLM graphs showing different degrees of patination observed on the archaeological specimens (magnification: 100X).

A group of small flakes with technological characteristics suggestive of lithic recycling^14^ (Fig. 1c) were studied (Supplementary Fig. S1). A sample of 283 such flakes (1–3 cm in length, on average) was selected and analyzed through an integrated approach based on four independent analytical techniques: (1) use-wear, (2) micro-residue analysis, (3) Fourier transform infrared (FTIR) microspectroscopy and (4) energy dispersive X-ray spectroscopy (SEM-EDX) (Supplementary Table S1). While chemical post-depositional patination partly limited the microscopic analysis of tool surfaces, macroscopic observations revealed minor or no evidence of mechanical stress, except for rare cases of edge-scarring attributable to the scattering of items in the sediment or excavation and post-excavation damages. These findings did not hamper the application of use-wear analysis mostly based on edge-damage identification. Scarring due to post depositional surface modification (PDSM) usually do no show the combination of features typical of items that have been used (edge rounding, edge morphology, edge location and distribution). PDSM are usually isolated, with uncommon shapes and distribution (Fig. 2a) and, in case of excavation damages, the edge appears freshly chipped with a different coloration from the surrounding patinated surface (Fig. 2b,c).

## Results

### Taphonomical processes affecting residue and use-wear preservation at the Revadim site

Sedimentological and micromorphological analyses conducted at Revadim revealed that the combination of at least four conditions permitted the preservation of organic and inorganic residues: 1. presence of water, 2. soil composition, 3. presence of heavy metals and carbonate, 4. soil pH.

#### Presence of water and soil composition

In general, water plays an important role in the preservation of residues. Although it is true that water-free environments are favorable for organic preservation because hydrolysis is not at work, waterlogged sites proved to preserve organic materials rather well^18,19^.

Sedimentological analysis performed at Revadim indicated that anthropogenic components have undergone post-depositional hydraulic winnowing which occurred several times throughout the entire sequence of deposition^20^.

In particular, it was established that long-term inundation characterized by high-energy flood and low-energy sheet wash occurred shortly after the deposition of layer C3^20,21^. Despite that, lithic artefacts in the sample studied are not abraded or heavily rolled and are relatively complete according to our microscopic observations.

Micromorphological analysis of Unit 2 showed that the sediments are composed of quartz with grain size ranging from silt to coarse sand, testifying that larger grains were deposited through water action (confirming the sedimentological analysis), while fine sand and silt were deposited either by wind and/or water. The quartz grains are bridged by a clayey groundmass that includes areas of micritic calcite. Thus, the source material of the palaeosol is mostly fluvial with some contribution of loessial dust^20^.

It has been argued that soils developed upon silt and clay substrates are characterized by a texture that inhibits water movement (allowing low hydraulic conductivity) by retaining it, largely preventing oxygen diffusion^22^. Thus, it can be assumed that soil composition along with prolonged water activity in layer C3 at Revadim allowed the formation of an anoxic environment, which usually characterized waterlogged contexts. Under these conditions, residues are more likely to survive. Within animal residues, adipocere will form rapidly on saturated or waterlogged soil, and it can form in a range of moist soil textures, including sand, silty sand, loam, clay, and sterilized soil. Adipocere is a result of chemical reactions and it can remain stable for a long time, able to survive, also in water, for thousands of years^23,24^.

#### Presence of heavy metals, carbonate and soil pH

Macroscopic and micromorphological observations, as well as micro-FTIR analyses, showed the presence in layer C3 of Unit 2 of abundant reddish-black nodules and crust composed of manganese and iron oxide minerals.

The water inundation in Unit 2 (see below) caused reducing conditions, and consequently, manganese and iron were mobilized. Following this process, soil pH increased, and oxides formed around nucleation centers^21^ (i.e., bone fragments).

Moreover, micromorphological observations showed that calcite infiltration into the palaeosol occurred after the deposition of manganese oxides, as confirmed by the lithic materials found on calcitic pendants in layers C2 and C3. This indicates that carbonate-rich solutions infiltrated from the overlying loess-derived Unit 1^21^.

A number of studies have demonstrated that the presence of heavy metals in sediment compositions can interfere with microbial activity and may guarantee preservation because biodegradation of organic components can be reduced by metal toxicity^18,25–27^. The presence of iron and manganese in Unit 2 Area C may have supported, to some extent, the preservation of residues. Moreover, the presence of carbonate-rich water percolating into Unit 2 surely facilitated the increase in soil pH. This condition allowed the preservation of bone residues, namely, the mineral part, which is known to be well preserved in soil with pH above 5.3^28,29^. Bone is mainly composed of the inorganic mineral hydroxyapatite, which is also the most durable and long-lasting component.

Soil pH can also affect adipocere formation: mildly alkaline soil is the most favorable^30^.

It is noteworthy that the most important (in terms of quantity and quality) type of residue found at Revadim is associated with hydroxyapatite.

Adipocere was also detected by FTIR in several specimens within the sample due to the specific and favorable taphonomic conditions reported below, which allowed its formation and preservation.

Conversely, the conservation of organic compounds related to animal residues is, in general, always more problematic.

Although site formation and taphonomic processes at Revadim played a favorable role in the preservation of ancient residues, its effect on the preservation of lithic materials was rather different. Affected by the same depositional and pedogenic conditions in Unit 2 layer C3, perishable organic and inorganic residues were able to survive, while flint implements became patinated.

Macroscopic and microscopic observation showed that all the analyzed items are characterized by a uniform sheen over their entire surface. At low magnification the surfaces appeared highly reflective but with no particularly pronounced smoothness while their physical coloration clearly changed from the original (mostly greyish) to a reddish/brownish shading with orange/yellow hints (Fig. 2d). The change in coloration was probably caused by mineral oxide (notably iron, known to be present in Revadim soil) and hydroxide that entered the flint via the absorption of moisture. Changes in flint coloration are commonly associated with *colour patina*, but physical characteristics of our sample seem to be closer to the *gloss patina* appearance^31–34^.

Even though at a macroscopic level the degree of alteration seems rather homogeneous, at high magnification it was possible to distinguish different degrees of intensity. The patination begins to affect the protruding point of the lithic surface in the initial stage of its formation until completely covering the surface with a characteristic gloss, having a bright and often pitted appearance (Fig. 2e–g). No striations were observed on top of the patina layer, which, together with the absence of rounding and fractures, led us to eliminate mechanical processes from the range of possibilities related to the patina formation. Indeed, geomorphological analysis in Area C showed that the burial of artefacts in layer 3 was relatively rapid and caused by low energy water action^20,21^.

We hypothesize that water activity, probably characterized by a specific pH (mostly alkaline^33^), was one of the main elements that contributed to the chemical reactions responsible for patina formation in Area C.

### Use-wear analysis

**Figure 3:**
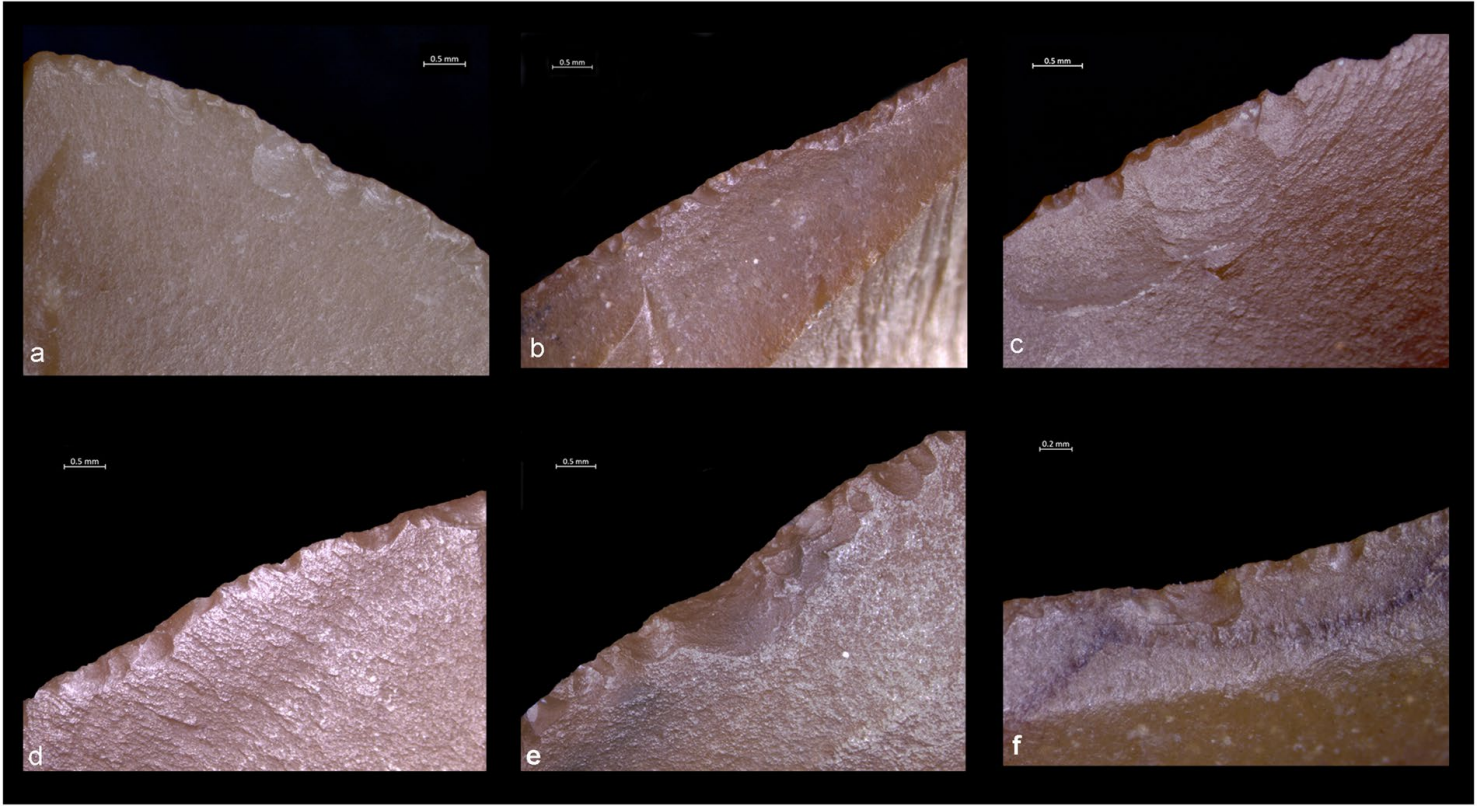
Edge-damage observed on small flakes from Revadim. (**a**) Scarring related to cutting soft to medium material. (**b**) Scarring related to cutting medium material. (**c**) Scarring related to cutting soft to medium material. (**d**) Scarring related to cutting soft to medium material. (**e**) Scarring related to scraping medium to hard material. (**f**) Scarring related to scraping soft to medium material.

Edge damage modifications resulting from use are evident in 38% of the entire sample (107 out of 283 analyzed specimens) and indicate that small flakes at Revadim were primarily used to work soft, soft to medium and medium materials by means of longitudinal motions (Supplementary Table S2). Forty-eight (48) items were interpreted as having had contact with soft materials, while fourteen (14) were used on soft to medium materials, thirteen (13) on medium materials, and one on medium to hard material. Edge damage related to these activities mainly consists of a combination of half-moon and cone scars with feather or step terminations. Their orientation is basically oblique and unidirectional while edge damage distribution, often developed only on one of the two faces, suggests a sharply inclined cut with flakes held at a 45° angle to the worked material (Fig. 3a–d). When the outer edge appears very thin and sharp, snapped edge areas with close and regular feather scars running inside it were observed. A low to medium degree of rounding related to cutting activities was also observed.

Transversal motions are less common within the sample, represented by fourteen (14) small flakes best suited for the processing of soft to medium and medium material (Fig. 3e,f). Edge damage is characterized by cone scars with mostly step and hinge terminations running perpendicular to the functional edge. Edge damage is localized along the ventral or dorsal surface, and the edge rounding is always pronounced.

Mixed actions were recognized on five specimens while three flakes were only interpreted based on the hardness of the worked material.

During the analysis we observed that small flakes were used to accomplish activities which did not require a change in the orientation of the artefact during manipulation, even though in one case a reversal of the artefact was required in order to change the edge portion to be used while direction of the motion was maintained.

The majority of the used flakes exhibited use-wear traces along one single edge, but in two cases, where more than one edge was suitable for use, we observed two functional areas which were utilized.

**Figure 4:**
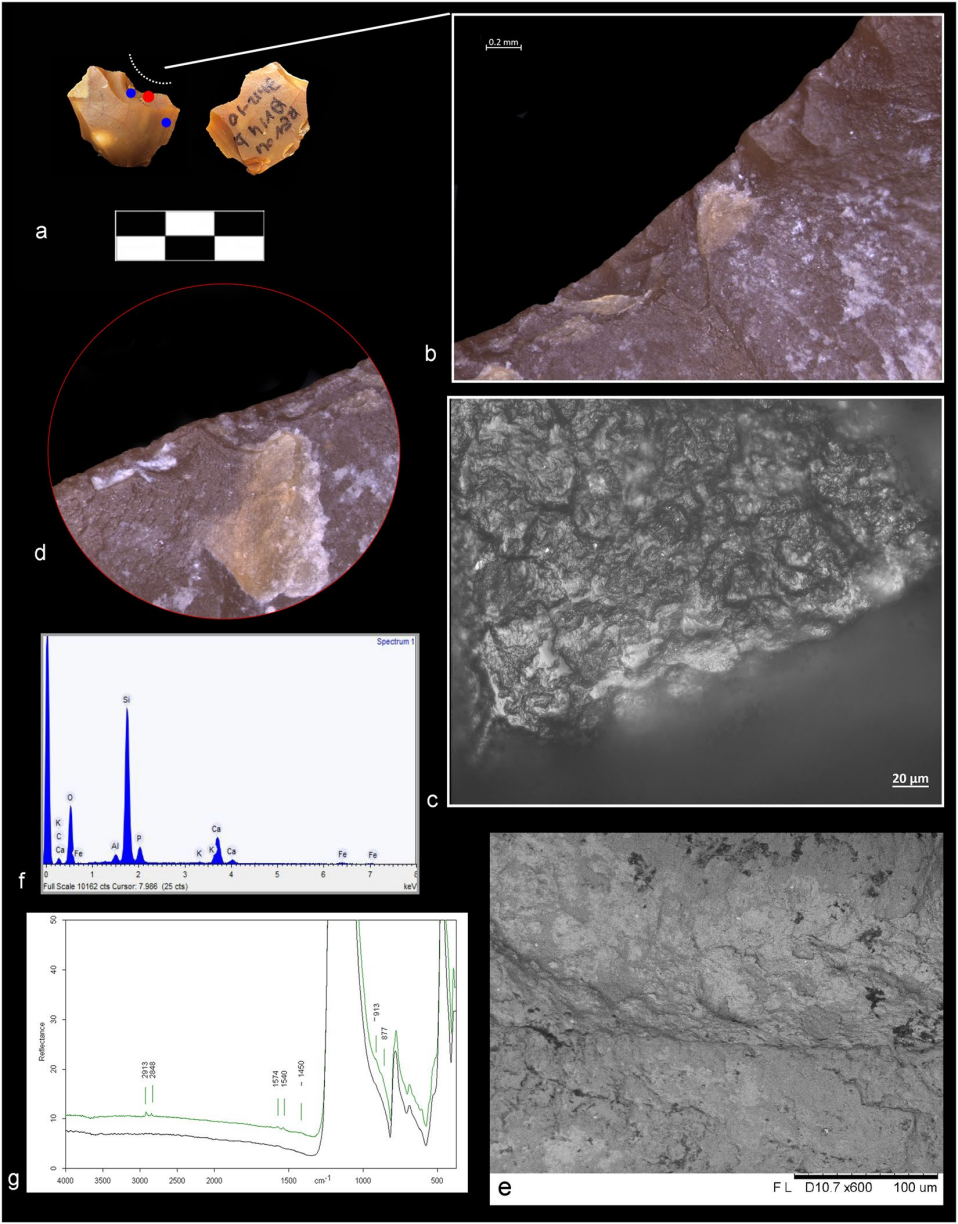
Double ventral lateral item with related use-wear and residue results. (**a**) Double ventral lateral item Av14b 71.12-10 #81. (**b**) Feather/step and hinge scars running along the outer edge with a transversal orientation and a close-irregular distribution associated with a transversal motion on hard material. (**c**) Bone-like polish located along the ventral edge and associated with prolonged contact with bone (magnification: 500X). (**d**,**e**) Close-up of the bony tissues entrapped on the damaged edge of the dorsal surface (OLM and BSE-SEM image). (**f**) EDX spectrum of residues on the active dorsal edge. (**g**) micro-FTIR spectrum of bone and adipocere micro-residues (green) over the dorsal surface. Black spectrum shows the fundamental mode of pure silica. Red and blue dots show respectively the EDX and FTIR sampling points.

Detailed microscopic observations, including micro-surface observations on five better-preserved items, showed evidence of contact with animal fleshy and connective tissues as well as sporadic or more prolonged contact with bone, which occurred during animal carcass processing. (Fig. 4, Supplementary Information and Supplementary Figs S2–S5).

### Residue analysis

**Figure 5:**
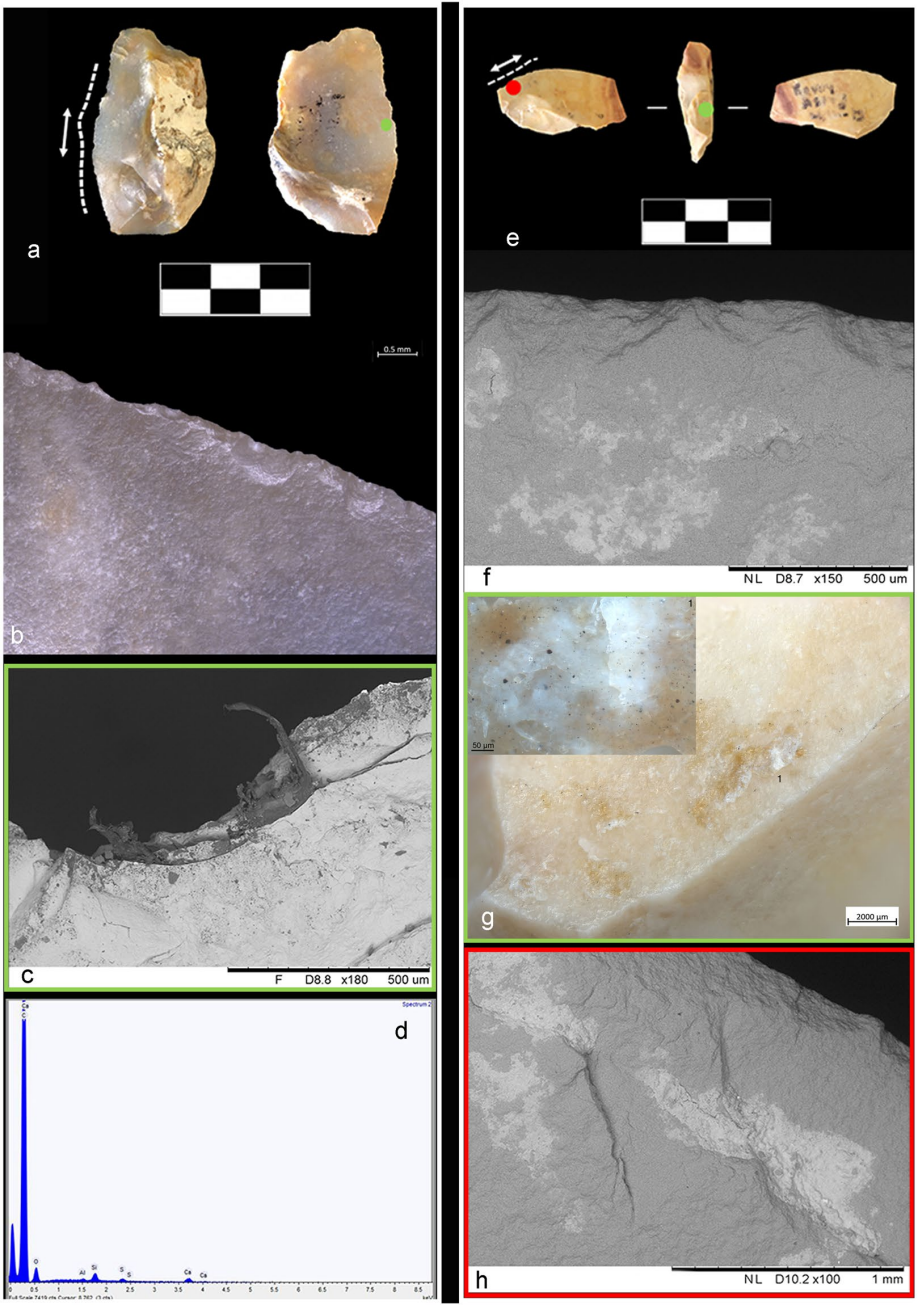
Example of animal residues found on small recycled flakes. (**a**) Double ventral lateral item AQ15a 71-12-08 #79. (**b**) Hinge and snap scars running along the outer edge with a close regular distribution and associated to a mixed motion on soft to medium material. (**c**) SEM image of collagen fibers smeared and entrapped in a wide scar along the used edge (BSE-SEM image). (**d**) SEM-EDX spectrum of the collagen fibers showing the characteristic peak of sulphur. (**e**) Double ventral regular item AS14d 71.14-13 #8. (**f**,**h**) SEM image of amorphous patches of white bone residues along the used edge (BSE-SEM image). (**g**) OLM image of white-yellow greasy amorphous structures compressed across a zone of prehension. In the close-up, note birefringent yellow collagen fibers entrapped inside the smeared residue.

**Figure 6:**
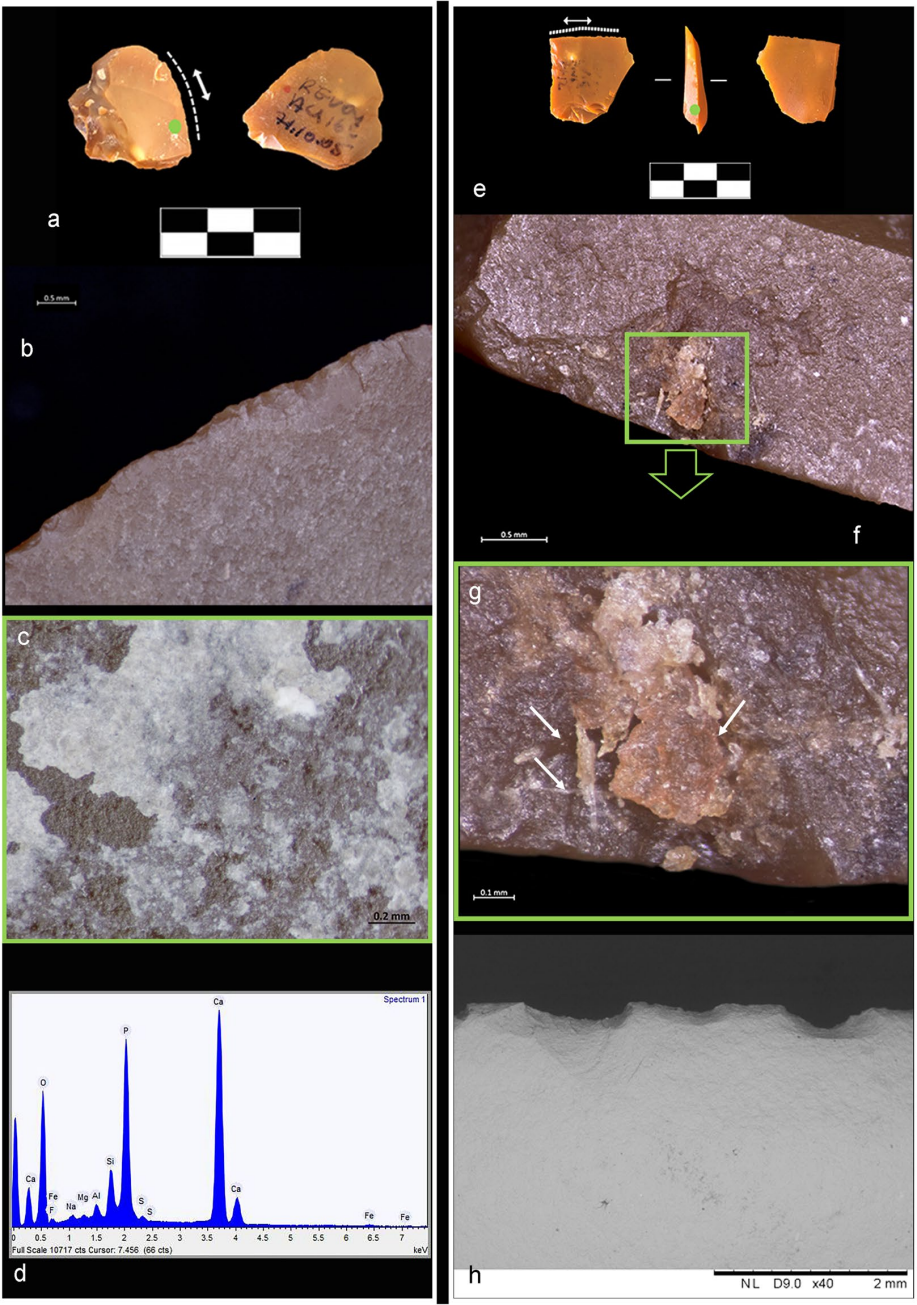
Example of animal residues found on small recycled flakes. (**a**) Double bulb Kombewa item AU16c 71.10-05 #62. (**b**) Feather and hinge scars running along the outer edge and interpreted as cutting medium-hard material. (**c**,**d**) Patch of compressed powder-like residue consistent with bony tissue and related SEM-EDX spectrum showing the diagnostic peak of calcium and phosphorous. (**e**) Double ventral lateral item AW16d 71.15-12 #88. (**f**) Greasy yellow-white amorphous animal residues compressed in a flint scar along the edge on the prehensile area and consistent with animal grease and fat. (**g**) Close-up of the same residual material showing fragments of birefringent collagen fibers entrapped inside. (**h**) SEM image showing hinge close-regular edge damage interpreted as cutting soft to medium material (BSE-SEM image).

Use-wear data is corroborated by the presence of different types of animal residues which were macroscopically identified on 11 small flakes. Figs 5 and 6 provide an example of four artefacts and related residues found entrapped inside flint scars (Figs 5c and 6c) along the edge as well as clustered and compressed on the zones of prehension (Figs 5g and 6f,g). Residues appear as patches of white and white-yellow greasy amorphous structures (Fig. 5f–h) as well as accumulations of yellow-brownish greasy matter with birefringent fibers clearly visible inside (Figs 5c,g and 6f,g).

To confirm the optical observations of residues, we conducted chemical and elemental analyses on 53 items using a SEM equipped with an EDX probe (Supplementary Table S3). Well-defined amorphous whitish masses of bone were identified on 30 small flakes (Supplementary Table S3). These are characterized by the two typical components of bone, calcium (Ca) and phosphorus (P), along with carbon (C), oxygen (O), and other trace elements (Fig. 4f, Supplementary Information and Supplementary Figs S2f, S4e and S5f). Moreover, micro-residues of animal tissues along the used edge were identified on four items. These residues are characterized by carbon (C), oxygen (O), sulphur (S), calcium (Ca), and other trace elements (Supplementary Information and Supplementary Table S3). These data are supported by the results of micro-FTIR spectroscopy on the active edge and on the prehensile areas of the flakes: the peak at 913 cm^−1^, attributed to bone, was observed on 22 items (Fig. 4g, Supplementary Information and Supplementary Fig. S5e and Table S3). In addition, the absorption band at around 1645 cm^−1^ was assigned to proteinaceous compounds related to collagen, while the doublet bands at around 1575/1536 cm^−1^ proved the presence of fatty acid salts (interpreted as adipocere, see Supplementary Information) on 27 artefacts (Fig. 4g, Supplementary Information and Supplementary Figs S4f and S5e). FTIR and EDX analysis showed a clear correspondence between use-wear and residue on 26 artefacts: animal residues were detected together with edge-damage due to use. Macro-residues of bony structures on 9 artefacts further support this analysis (Supplementary Table S3).

**Figure 7:**
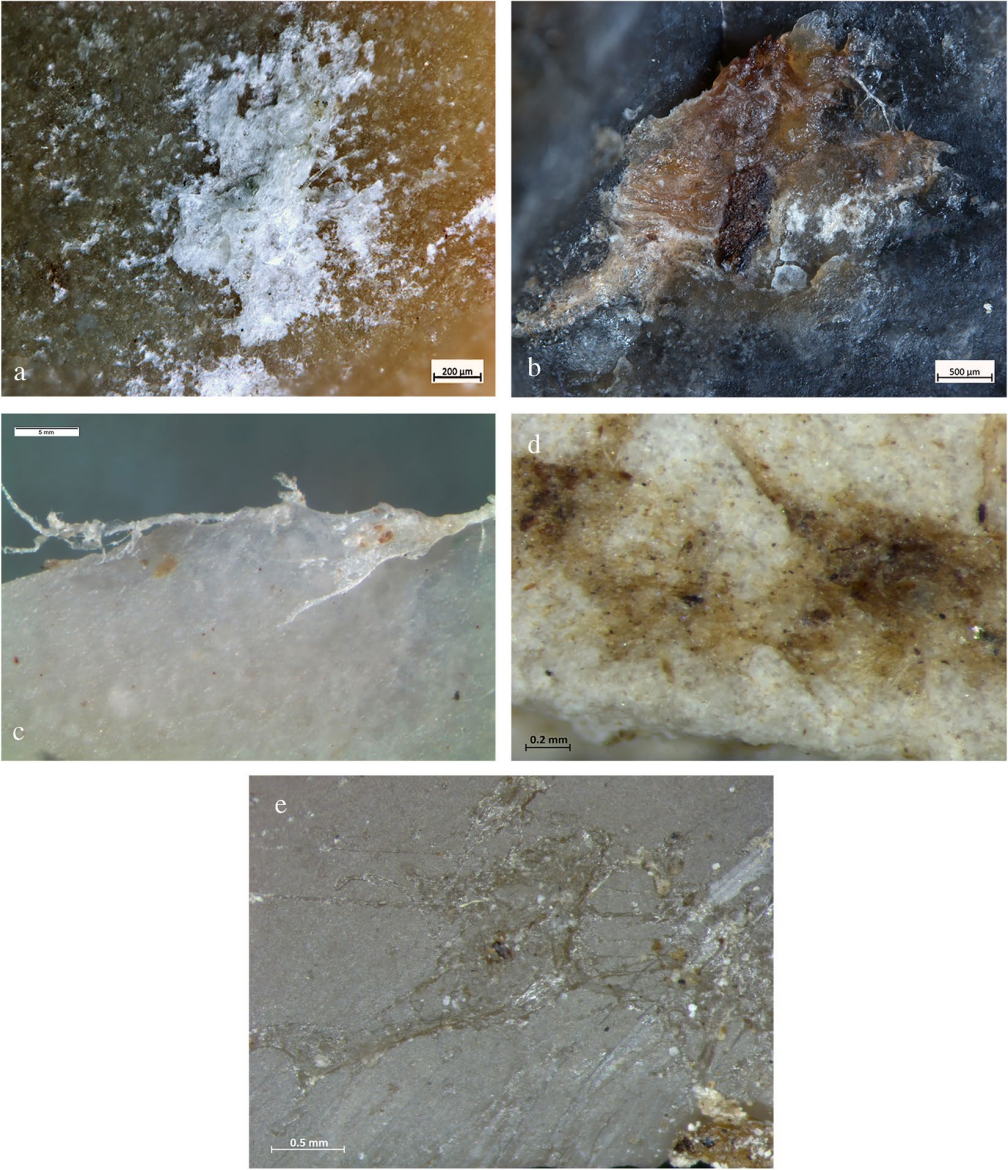
Experimental residues after processing animal materials. (**a**) Patch of bone powder and collagen fibers from experimental periosteum removal. (**b**) Experimental yellow-reddish animal residue of fat, blood, and collagen fibers localized away from the edge of a tiny tool used in butchery. Notice the greasy, “mud-cracked” and desiccated appearance of the residue. (**c**) Elongated fibers left after butchery activity along the edge (**d**) Compressed animal residual matter on the cortical prehensile area after filleting meat. (**e**) Close-up on fat residual matter smeared far away from the edge of a small tool used for skinning hide.

Use-wear and residues identified on the small flakes were interpreted by comparison with an extensive experimental reference collection of replicas that we tested on animal carcasses (Fig. 7; Supplementary Information and Figs S6–S8). Our butchery trials showed that small flakes are particularly suitable for performing accurate and brief gestures on small to medium sized animals or animal body parts. We performed several butchery experiments on small and medium carcasses (e.g., wild boar, roe deer, and sheep) and found that small flakes were very efficient for fine cutting activities aimed at removing the hide during the skinning process, filleting meat, stripping meat from bone, and scraping off periosteum tissue and cartilage to facilitate bone breakage. Experimental data produced by other researchers^35–37^ also highlight the suitability of small flakes for tasks requiring precision and *finesse* while applying little force for short periods of time on material that is not very thick.

## Discussion

Using an integrated experimental framework (see Supplementary materials) and a use wear and residue study, we were able to identify the role small flakes played within the entire butchery process. We argue that the inhabitants of Revadim processed carcasses for food acquisition in layer C3 using the small flakes purposely produced in that context. The small flakes were characterized by the recurrent and significant presence of fat, bone tissues, and collagen fibers. This, along with the limited use of the edge portion of the flakes, demonstrates the direct connection of these artefacts to specific gestures at precise stages of the butchery sequence, probably when the carcass was already partially dismembered and/or defleshed. There are in fact specific butchery tasks that can be easily performed with small recycled flakes, while the use of small flakes in other tasks seems less efficient. As testified by our experimental trials, the morphological characteristic of these flakes (thin and sharp edges, small dimension, limited used edge portion, fast production process) make them perfectly suited for fine scale activities where the control of the gesture and the precision of the motion are decisive. Within a butchery process, these tasks include deskinning, muscle cutting and filleting of meat, cleaning bone from meat and removing the periosteum for assist the bone breakage, cutting tendon and ligaments. We noted that the maximum yield of these tools is expressed when the materials processed are limited in size and/or volume and when the need for accuracy is the primary objective sought. At the same time, the small recycled flakes are not multifaceted or flexible type of tools. Activities such as sawing bone, scraping hide, dismembering large carcasses are very difficult, rather impossible, to conduct with small implements. Heavy-duty butchery tasks, such as dismembering and disarticulating, were probably performed with larger, heavier tools (bifaces, larger flakes, or other tool types) with longer cutting edges and with an increased loading potential. Although small animal carcasses may be easily butchered with small recycled flakes, from the beginning of the process until the end, we do not believe that their use was exclusively addressed in such tasks. We rather suggest that, especially in the case of medium-large carcasses, LCTs and small flakes acted together within the same butchery sequence and that their utilization was well planned by the Revadim hominins which were aware of the perfect time and situation to use the one or the other. As Key^38^ argues, there is a direct correspondence between the form and the function of lithic tools that might explain why past human populations produced tools of specific sizes and why tool size varied within and among artefact assemblages. Butchery experiments using tools of different morphology and size demonstrated the efficacy of these sets of tools in specific tasks^39–41^. Therefore, we argue that Lower Paleolithic humans stripped carcasses using diversified tool-kits created to meet their required needs in each stage of the butchery sequence.

The production and use of small flakes and the presence of butchered animal remains, unearthed together at several Lower Paleolithic sites in Africa, Asia and Europe^6,12,13,42,43^, suggest an intriguing relationship. The archaeological record provides ample evidence that carnivory based on hunting was one of the greatest driving forces in human biological and cultural evolution, starting as early as 2 million years ago^44,45^. The frequency of exploited animal remains bearing cut-marks and percussion marks supports this view, as do use-wear studies indicating that small flakes were hand-held tools used in animal butchery tasks that required precision^6,12^. Our findings show a clear relationship between the production and use of tiny flakes and the need to obtain meat and fat for adaptive purposes, which, alongside the use of larger tools, may constitute a behavioral marker of late Lower Paleolithic lifeways. We therefore see the production and use of small flakes as one of the significant transformations in early human life that took place some 500 kya, along with changes in environment, faunal species, lithic technologies, and human lineages^46–48^.

## Materials and Methods

### Materials

The small flakes presented here were produced from previously manufactured and discarded flakes which were transformed into cores. These “old” flakes are termed here Cores-On-Flakes/Flaked-Flakes^14^ (henceforth COF-FFs, Supplementary Fig. S1). Such items at Revadim fit the definition of “flaked-flakes” defined by Ashton^49^ and are different from Middle Paleolithic cores-on-flakes^50–55^. In total, 944 COF-FFs and 708 blanks produced from COF-FFs were detected within the lithic assemblage of layer C3.

The COF-FF category includes “parent flakes” that had small flakes removed from their ventral or dorsal faces (or both), proximal or distal ends (or both), and lateral edges. Removals were most commonly produced in a straightforward manner, with or without platform preparations.

Most of the COF-FFs are characterized by the production of small flakes from the ventral face of the parent flake (Supplementary Fig. 1a). COF-FFs with a single ventral removal constitute 29.0% of the COF-FFs (n = 274), while COF-FFs with multiple removals from the ventral face constitute 26.0% (n = 246). These are followed by COF-FFs with combined removals from both the ventral and dorsal faces (n = 179, 19.0%; Supplementary Fig. 1b) and they are divided into two sub-types: COF-FFs with removals unrelated to each other (n = 108) and COF-FFs with removals some of which were used as a striking platform for others (n = 71). An additional 17.2% of the COF-FFs have a single dorsal removal (n = 162, Supplementary Fig. 1c). Only two items (0.2%) resemble the truncated-faceted technique^53^.

Blanks produced from COF-FFs (BPFCs, Supplementary Fig. S1d) present a wide variety of types. Double ventral flakes that cannot be assigned to a specific category (‘varia’) are the most common type, with 183 items (25.9%), followed by double ventral regular flakes (n = 163, 23.0%), double ventral reversed lateral flakes (n = 119, 16.8%), and double ventral lateral flakes (n = 118, 16.7%). Other sub-categories appear in lower proportions, however still in noteworthy quantities, demonstrating the different modes of production of small flakes from parent flakes^14^.

One-fifth of the BPFCs (n = 148, 20.1%,) present patina on their dorsal faces, and post-patina surfaces on their ventral face. These items demonstrate a two-stage life history: the original flake was produced, then covered by patina, and afterwards was used as a core-on-flake for the production of a small sharp flake. Such patinated items with post-patina removals are present in all sub-categories. The presence of patina and post-patina removals indicate a time gap between the two stages of use^56^, suggesting, in these cases, separate procedures of manufacture and recycling, rather than the existence of a single *chaîne opératoire* (as in the case of ramification^57,58^). Moreover, a wide variety of blank types were selected to be used as COF-FFs, implying that these blanks were not originally produced for that purpose but rather were selected out of the many existing artefacts manufactured by different lithic production trajectories practiced at the site. We therefore argue that the small flakes found at Revadim and studied here were produced by means of lithic recycling^14^.

Indeed, not all blanks used as COF-FFs bear post-patina removals^14^. However, these items reflect a change in function, and therefore demonstrate the existence of two life-cycles. We therefore suggest that most of the COF-FFs and BPFCs reflect a process of lithic recycling.

While some scholars^59^ argue that flakes produced from COF-FFs are too small to be used, other authors suggest that such small flakes should be viewed as practical artefacts^51,60^. Indeed, the efficiency of small flakes was demonstrated in the past^36,61^. Use-wear studies provide further support for such notions, indicating the effective use of recycled small flakes^35,60,62^.

The deliberate production of tiny flakes from earlier Lower Paleolithic contexts such as Ubeydia^63^, Fuente Nueva 3^64^, and Bizat Ruhama^65^ implies a continuous manufacture of small flakes throughout the Lower Palaeolithic period. Our data, however, suggest an intensification in the manufacture of such small flakes during the Late Acheulian in the Levant, a trend that seems to continue into the Acheulo-Yabrudian cultural complex^35,55,66,67^.

## Methods

### Technological analysis

The analysis of the lithic assemblage of layer C3 includes 28,059 items^14^. All items described in this paper as a part of the recycling phenomenon (*i*.*e*., COF-FFs and BPFCs) were typologically and technologically classified in a previous study^14^. In addition, the presence of patinated surfaces was indicated. The original blank of each COF-FF was also indicated.

In addition, a random sample of 150 of all COF-FFs from C3 (~16% of the COF-FFs), and 100 of all the blanks produced from COF-FFs (BPFCs, corresponding to ~15%), were arbitrarily selected and measured for metrics (length, width, thickness and weight)^14^.

### Use-wear analysis

Small flakes produced by means of lithic recycling were analyzed using Low- and High-power approaches^34,68–70^. The combination of the two methods allows observing the use-wear evidence at different magnification.

In our work, a Zeiss Discovery stereomicroscope with a zoom up to 8x, objective 1x, oculars 10x and equipped with a LED ring-light, was used for edge damage characterization, and a Nikon Eclipse metallurgical microscope in reflected light, capable of magnification up to 500x, was used for micro polish characterization. The low magnification pictures were taken with a Zeiss Axiocam 105 Color camera directly connected to the stereomicroscope, while a digital camera ToupView connected to the metallurgical microscope was used for the micro use photographs. All picture settings were chosen in the course of the microscopic analysis, using dedicated software. Finally, the Helicon Focus Software was used to extend the depth of field.

Edge damage was described according to characteristics such as initiation, termination, direction, location, and distribution. Edge rounding was defined by its degree.

Micro wear such as polish was described based on its features. Polish was interpreted based on its texture and topography along with its orientation and distribution.

The surface micro rounding was described according to its degree.

### Residue morphological analysis

Residue morphology was analyzed at DANTE - *Diet and Ancient Technology Laboratory*, at Sapienza University of Rome using a stereoscope Zeiss Axio Zoom with magnification ranging from 10x to 178x and a metallographic microscope Zeiss Axio Scope A1 with magnification ranging from 10x to 400x.

Archaeological residues were identified, described, and interpreted according to their appearance, morphological features (color, inclusions, consistency, birefringency, etc.), and spatial patterns of distribution^18,71^. Interpretations were suggested on the basis of a comparison with a collection of experimental residues produced by using stone tools in different activities (butchering, bone, hide processing, etc.) as well as by comparison with available literature discussing archaeological as well as experimental residues on stone tools^72–81^.

### Fourier transform infrared micro-spectroscopy

The Fourier transform infrared (FTIR) microspectra were collected with a Bruker Optic Alpha-R portable interferometer with an external reflectance head covering a circular area of about 5 mm in diameter. The samples were placed directly in front of the objective, without preliminary treatments, and different spots were selected for analysis. The investigated spectral range was 7000–375 cm^−1^ with a resolution of 4 cm^−1^, and at least 250 scans were performed.

Infrared measures were taken on specimens at least on 3 points (proximal, medial and distal) along the dorsal and ventral surface according to the used edge, and at least on 2 points on the inner dorsal and ventral surface of each item.

### Scanning electron microscope and energy dispersive X-ray analysis

The electron dispersive X-ray (EDX) analysis of the experimental and archaeological stone tools was performed with a Hitachi TM3000 scanning electron microscope (SEM) equipped with a SwiftED3000 energy dispersive X-ray spectrophotometer. Although flint tools are non-conducting specimens, a coating procedure with conductive materials (e.g. carbon, gold) is usually required. In our case, specimens were not coated and the “charge-up” phenomenon was avoid by imaged specimens within the magnification range of 500–800x. Different accelerating voltages were used during the analysis of residues: 5 Kv was used to characterize topographic and textural traits while the 15 Kv mode provided elemental information through grey-scale images according to the atomic number using the high sensitivity backscattered electron detector.

Electron dispersive X-ray spectroscopy was performed on each identified residue with 2 or 3 measurements taken on different spots of the same residue at 15 kV accelerating voltage in BSE mode with a magnification from 500x to 800x and an acquisition time of 400 s.

The methodological protocol for use-wear and residues included the use of powder-free sterile gloves during the manipulation of cleaned objects and the use of a Parafilm (laboratory film) to wrap the modelling clay supporting the pieces during the observations.

### Cleaning protocol

The archaeological sample was observed with the naked eye and under a stereomicroscope in order to preliminarily identify edge damage and residues. Edge damage and residues related to use were photographed, mapped, and documented before and after cleaning procedures.

The samples that underwent spectroscopic (FTIR) and elemental analyses (SEM-EDX) were washed in deionized water in an ultrasonic tank for 10 minutes just before the analysis.

Afterwards, in order to carry out the functional analysis and to eliminate contamination originating from the manipulation of the artefacts during classification, the specimens showing use traces were gently washed under tap water and then soaked for 10 minutes in an ultrasonic tank with deionized water.

The experimental specimens were quickly washed under tap water before residue analysis. Patches of residues were still firmly attached on the lithic surface after this procedure and were morphologically and chemically analyzed and recorded.

In order to carry out the microscopic observations, the replicas were placed in a chemical bath starting with dilute 3% acetic acid (CH_3_COOH) lasting 12 min followed by dilute 3% sodium hydroxide (NaOH) for another 12 min. Finally, the objects were washed with deionized water in an ultrasonic tank for 10 min.

## Supplementary information

Supplementary Information

## Data Availability

Data discussed in this paper can be found in the Supplementary Information.
