# Supplementary material for: Animal residues found on tiny Lower Paleolithic tools reveal their use in butchery
Source: Sci Rep. 2019 Sep 10;9:13031. doi: 10.1038/s41598-019-49650-8 (PMC6736941; doi:10.1038/s41598-019-49650-8)
Supplement: Supplementary file 1 — Supplementary Information [file 41598_2019_49650_MOESM1_ESM.pdf]

Animal residues found on tiny Lower Paleolithic tools reveal their use in butchery

Supplementary Information

Flavia Venditti[1,4*], Emanuela Cristiani[2], Stella Nunziante-Cesaro[3], Aviad Agam[1], Cristina Lemorini[4], Ran Barkai[1]

[1]Institute of Archaeology, Tel Aviv University, Tel Aviv 69978, Israel.
[2]DANTE Diet and Ancient Technology Laboratory, Sapienza, University of Rome, Via Caserta 6, Rome 00161, Italy.
[3]Scientific Methodologies Applied to Cultural Heritage (SMATCH), ISMN-CNR c\o Dept. of Chemistry, Sapienza, University of Rome, P.le Aldo Moro, Rome 00185, Italy.
[4] Department of Classics, LTFAPA Laboratory, Sapienza, University of Rome, P.le Aldo Moro, Rome 00185, Italy.
*Corresponding author: flavia.venditti@gmail.com

1. Regional setting

Revadim is an open-air site located on the southern coastal plain of Israel, 40 km southeast of Tel Aviv. Four seasons of excavations were conducted during the years 1996-2004 on behalf of the Israel Antiquities Authority and the Hebrew University of Jerusalem[17,21].

The site was preliminarily dated by palaeomagnetic analyses of the geological sequence, demonstrating normal polarity and thus indicating that the entire sequence is younger than 780 kyr[17,82], and by uranium series dating of carbonates covering flint artefacts, yielding dates between 500-300 kyr[20]and providing a minimum age for these artefacts. Given the characteristics of the lithic and faunal assemblages, the entire anthropogenic assemblage was assigned to the Late Acheulian cultural complex of the Levant[15,16,21,83].

The excavations at the site were conducted mainly in Areas B and C. In total, seven archaeological layers were exposed[83]. Area C is divided into two sub-areas: C East and C West, located 8 m apart[83]. In Area C West, which covers an area of 33 m^2, five superimposed archaeological layers were exposed, labelled C1 to C5, from top to bottom[83]. Layer C3 in Area C West, the focus of this current work, is the densest layer at the site, both in terms of flint artefacts and bones[84].

Revadim's faunal assemblages include thousands of animal bones, with a pronounced presence of *Palaeoloxodon antiquus, Bos primigenius*, and *Dama* cf. *mesopotamica*, in addition to other mammalian

species and micro vertebrates (for more details see[15]). The presence of *Palaeoloxodon antiquus* in Revadim may imply a wide range of environments, from wooded to more open areas[21].

Use-signs and fat residues on a handaxe and a scraper from Area B were found in association with butchered elephant remains[16]. These results provide some of the earliest direct evidence for meat and hide processing as well as meat consumption by early humans.

2. Use-wear and residue results

The description of the use-wear and the related residues of the five better-preserved items is provided below (for technological definitions see[14]):

-Double-bulb Kombewa item AV15b 71.09-06

This item is 1.8 cm long, 2.6 cm wide, and 0.8 cm thick. It weighs 3 grams and it was flaked without any platform preparations. It is in an average state of preservation showing a uniform light degree of sheen affected the protruding points of both the ventral and dorsal surfaces.

Edge damage testified to a longitudinal motion on a medium material attested by slightly oblique half-moon sliced scars, run together along both the edge surfaces with a close-regular distribution (Supplementary Figure S2b). Microwear is represented by rough granular polish which appears as smooth and domed on specific spots caused by a more prolonged contact with a hard material (Supplementary Figure S2c). Polish distribution may be considered spotted, mostly due to the alteration which prevented the observation of the polish continuity along the outer edge. The tool was interpreted as having had a sharp and intense longitudinal contact with fresh bone, which was probably still covered by meat at the time of its processing. In fact, observations made with SEM-EDX confirmed the presence of micro-residues of bone (showing several whitish patches of the mineral hydroxyapatite) together with a peak of sulphur, testifying to the remaining presence of proteinaceous materials (Supplementary Figures S2e,f).

FTIR analysis detected a band at around 1580 cm^{-1}, which does not correspond to any chemical bond detected experimentally.

-Double ventral varia item Aw14c 71.14-12

The item is 4.0 cm long, 3.2 cm wide, and 2.0 cm thick. It weighs 18 grams. It was flaked from a modified base, cutting the distal edge of the original ventral face.

The flake showed a heavy degree of alteration on its dorsal surface characterized by a covering glossy patina while on the ventral surface the alteration was present but less developed.

The edge damage is represented by hinge and step termination scars running along the dorsal and ventral edge characterized by a close-regular distribution and a semi-oblique orientation (Supplementary Figure S3b). Because of the diffused alteration traces, the polish was observed only on a specific spot along the ventral edge, between the intersections of the distal with the lateral edges. Polish appears rough to smooth with a domed topography (similar to a hide-like polish) more developed along the outer edge (Supplementary Figure S3c). The polish was too limited to characterize its orientation.

The item was interpreted to be used during an activity involving a contact with fresh animal material of a medium hardness (e.g., cartilage) and probably hide through a longitudinal motion.

The FTIR analysis only detected generic C-H bonds, which are not sufficiently diagnostic to provide information concerning the nature of the residues, and no micro residues were detected through the EDX analysis or through morphological observations.

-Double ventral regular item AQ17b 71.08-01

This item is 0.6 cm long, 1.7 cm wide, and 0.2 cm thick. It weighs 1 gram. The flaking of the item was performed without any platform preparations, from a cortical edge.

The flake was considered suitable for microscopic observation even though its dorsal surface was affected by a heavy glossy patina totally covering the surface, while the ventral surface appeared preserved.

This flake is the smallest used flake within the sample. Its functional edge corresponds to a small rectilinear portion of its lateral side, which was fractured before use. On this spot, edge damage on the dorsal surface exhibited cone feather features along with the outer edge with a close-regular distribution and a transversal orientation (Supplementary Figure S4b). Edge rounding is absent. Micro wear along the dorsal edge showed a bright rough and granular polish with a discontinuous distribution running from the outer edge to the surface (Supplementary Figure S4c). We interpreted the item as presenting a gently transversal contact with fresh

fleshy tissues for scraping off the meat from the bone. Adipocere was identified along the dorsal functional edge through FTIR micro-spectroscopy (Supplementary Figure S4f), while EDX analysis proved the presence of hydroxyapatite attested on a spot along the dorsal edge represented by peaks of calcium and phosphorous at the ratio 2:1 (Supplementary Figures S4d,e).

-Double ventral lateral itemAv14b 71.12-10

The item is 2.3 cm long, 2.5 cm wide, and 0.7 cm thick. It weighs 4 grams. It was flaked without any platform preparations.

The state of preservation of the artefact is characterized by a uniform reddish-brown color patina with a medium degree of sheen displayed at a microscopic level.

This item is characterized by an excellent correspondence between use wear and residues. Edge damage is represented by a combination of feather/step and hinge scars running along the outer edge with a transversal orientation and a close-irregular distribution (Fig. 4b). A medium to high degree of edge rounding affected the tool's edge. Because of the poor state of preservation of the flint surfaces, only a spot of polish was observed inside a hinge scar along the ventral edge characterized by a well-developed smooth and domed to flat (bone-like) polish topography (Fig. 4c). Macro and micro wear traces suggest an activity using a transversal motion on hard animal materials involving repetitive and prolonged contact with bone. A dense white amorphous residual deposit consistent with fat was detected still entrapped in a crack along the dorsal edge while whitish bony tissues were identified inside the edge scar, on the ventral surface (Figs. 4d,e).

Bone micro-residues were jointly confirmed by micro-FTIR and EDX analyses, as was the presence of adipocere, at the frequency ~1574/1540 cm^{-1} on the dorsal edge and on the inner dorsal surface (Figs. 4f,g).

-Double ventral lateral item AS14a 71.11-08

The item is 2.3 cm long, 1.3 cm wide, and 1.3 cm thick. It weighs 3 grams. The item was flaked without any platform preparations. The original ventral face and the actual ventral face have slightly different texture.

Several areas of the artefact were affected by a medium to high degree of glossy alteration on its dorsal and ventral faces, although some portions of the flint surface were affected to a lesser extent.

Edge damage is represented by feather scars becoming step with a close-regular and overlapping distribution (Supplementary Figure S5b). They are located in the central portion of the used edge where the material was probably impacted the most. Their orientation is mostly transversal.
Polish is visible on the ventral face and it is characterized by a rough to smooth texture and a granular to domed topography with a greasy appearance (Supplementary Figure S5c). The edge is affected by a high degree of rounding. The tool is interpreted as having been used to process animal fleshy tissues including fat and involving contact with bone during the processing of animal carcasses. Micro-residues are morphologically identified as whitish/yellowish bony tissues smeared far from the edge towards the end of the tool (towards the bulb) and with a straight distribution typical of fresh material (Supplementary Figure S5g). Bone residues were confirmed by chemical analyses through FTIR and EDX (Supplementary Figures S5e,f).

2.1 Chemical results

Fifty-three (53) specimens out of the one hundred and seven (107) used implements were subjected to FTIR and SEM-EDX analysis (Supplementary Table S3).

Using Fourier Transform Infrared micro-spectroscopy (micro-FTIR), we were able to identify preserved micro-residues on forty specimens (40).
Bone micro remains were detected on the working edges as well as on the inner surfaces on twenty-six (26) items. Bone is present in the form of hydroxyapatite crystals, the inorganic mineral component of bone tissues. The hydroxyapatite is represented on micro FTIR spectra by a shoulder on the low frequency side of the Si-O stretching mode at ~913 cm^{-1} corresponding to the PO_3= stretching mode of calcium phosphate[28,85] (apatite, Fig. 4g and Supplementary Figure S5e). In 17 cases, typical doublet absorption bands at the frequencies 1574/1540 cm^{-1} were also detected, indicating the presence of fatty acid salts (palmitate and/or stearate) composing the adipocere (Fig. 4g and Supplementary Figures S4f and S5e).
In the spectra of eight flakes only the mentioned doublet was observed together with the C-H stretching modes around 3000 cm^{-1}. Adipocere is physically described as a durable wax-like organic matter, mostly colorless, whose preservation in archaeological contexts depends on specific environmental conditions (e.g. humidity).

It is formed as a result of bacterial activity converting fats into a mixture of palmitic, stearic and myristic fatty acids, and/or in their calcium salts[86,87].

Moreover, on eight (8) spectra we found a band at ~1640 cm^{-1} in association with peak and bands related to bone and adipocere absorptions. This band is assigned to the amide I ($\nu_{C=O}$) mode, which is the most intense absorption band of collagen, the first structural protein in various connective tissues in animal bodies[28,85,88-91]. (Supplementary Figure S4f).

In addition, when animal residues were recorded, a C-H stretching modes at ~2916 and ~2848 cm^{-1} always supported the presence of organic compounds.

These fifty-three (53) specimens were also analyzed using an EDX probe mounted on a Scanning Electron Microscope (SEM) in order to 1) provide high-resolution topographical residue details, and 2) determine the elemental composition of residues (Supplementary Table S3).

Bone micro residues were identified on different areas of the working edge on 30 items as well as on the inner tool surfaces associated with the prehensile areas. Bone micro residues appeared at high magnification as amorphous whitish patches with a rough and sometimes cracked appearance spread on the micro-cavities of the flint structure (Supplementary Figures S2e and S4d). The mineral part of bone, the hydroxyapatite, resulted in a combination of calcium (Ca) and phosphorous (P) at the ratio 2:1[81,92](Fig. 4f and Supplementary Figures S2f; S4e and S5f).

On three (4) items, fleshy tissue remnants and a collagen fiber (Fig.5c) were observed in association with bone micro remains. They appeared through SEM as smeared amorphous black patches of animal matter (a mixture of superimposed portions of fleshy tissues and organic components) characterized by a peak of sulphur (S) and phosphorous (P). On two out of the three items, this data was confirmed by the ~1640 cm^{-1} proteinaceous band on the infrared spectra.

The integration of the two techniques confirmed the presence of bone micro-residues on seventeen (17) items out of fifty-three (53) analyzed items while on twenty-seven (27) specimens, the EDX and FTIR results both concur with the presence of animal residues through the detection of bone, adipocere, and proteinaceous compounds (Supplementary Table S3).

3. Experimental framework

In accordance with the archaeological data, we focused our reference collection on the exploitation of animal carcasses, performing two complete butchery trials on two medium size animals: a roe deer and a juvenile deer (Supplementary Figure S6a). Animals were butchered after they died of natural causes (including an accident) and carcasses were processed courtesy of the Parco Nazionale d'Abruzzo, Lazio e Molise.

Three small flake replicas (2 lateral and a Kombewa flake, Supplementary Figure S6d, blue square) were used through all the main stages of the butchery sequence, starting from the skinning process, through the dismembering, the disarticulation, the filleting of meat, and the stripping of flesh from bones (Supplementary Figures S6a2-4).

Since the archaeological specimens showed a significant presence of fresh bony tissue and fat residues on their surfaces, we also include in the reference collection the processing of fresh bones (cow femurs) with the aim of removing the periosteum before bone breakage for marrow extraction (Supplementary Figure S6a5).

In addition, our observations and considerations relied on a rich collection of experiments produced in recent years by FV, where dozens of small flake replicas were used for processing animal materials and testing different stages of the butchery sequence (Supplementary Figures S6a6-8 and S6d, red square).

This *corpus* of experiments allowed us to: 1) validate our interpretations of the development of the use wear traces and related residues; 2) test the efficiency of the recycled small flakes during the different stages of the butchery process.

The experimental replicas were produced following the same technological procedures recognized for products of recycling at the site (Supplementary Figure S6d).

3.1 Use-wear results

According to Key and Lycett[36], there is a threshold below which flakes of specific size (small, in our case-study) become inefficient in particular working conditions. A previous experiment (FV's reference collection) performed on a carcass of an adult male wild boar highlighted the efficiency of the small flakes in performing specific activities, while for other activities they were found to be unsuitable. The skinning process was rather easily performed with tiny flakes, which displayed good cutting efficiency thanks to their sharp edges. The

disarticulation of the hind leg, however, could not be performed by these small flakes due to the difficulty in controlling the penetration of the cutting edge into the great amount of meat surrounding the bones and joints. Moreover, the small dimension of the two used tiny flakes prevented comfortable manipulation, making the gripping very slippery.

Following these results, another butchery experiment was specifically organized to focus on the butchery of a small-medium animal (a roe deer and a juvenile specimen of deer). Due to the small size of these animals, the amount of fresh tissue was limited, and the animal skeletal composition was relatively easy to work with small flakes even for inexperienced butchers. In these two experiments, small flakes used demonstrated to be suitable for skinning, filleting meat, and stripping flesh from bone. The dismembering, which involves the disarticulation of the joint, was rather hard to perform due to the difficulty of penetrating into the joints to cut ligaments and tendons. In contrast, the small flakes were perfectly suited to activities that required precision and control to work materials that did not require significant loading force (e.g., removing of the periosteum by scraping, cutting fresh meat, cutting fresh hide, removing meat from bone). We performed mainly longitudinal motions, although longitudinal and transversal motions might have been performed interchangeably during the entire butchery sequence.

From a functional point of view, edge damage and microwear associated with a butchery process performed with small flakes are characteristic of both soft/medium and medium to hard materials. Soft to medium materials, usually represented by meat, fat, hide and tendons, are associated with feather, half-moon and step scar terminations along with snap fracturing, which may include smaller feather removals inside (Supplementary Figures S6b1-8). Edge rounding is almost never highly developed except for very thin and acute edges, which round quickly, especially after working hide. Micro polish is characterized by a bright greasy appearance with a rough to smooth texture and a granular towards domed topography (Supplementary Figures S6c1-8). The freshness of the worked materials is reflected in the greasy and bright polish appearance. Bone was frequently hit throughout the butchery activity, resulting in hinge and/or step scars with a discontinuous distribution along the used edge (Supplementary Figures S6b2,4). At a microscopic level, an easily recognizable bone-polish with a smooth texture and a domed to flat topography is apparent (Supplementary Figures S6c3,4). Since the contact with bone was mostly accidental, the distribution of the polish along the functional edge is spotted and well-developed edge rounding is observable only on flakes used

to scrape off the periosteum. In this case the edge rounding is due to the repetitive and more prolonged contact with the hard material. When bones are processed for removing the periosteum, the presence of both soft materials such as fresh fat and meat along with the harder bone structures and connective tissues allows the development of traces associated with both classes of material. As shown in Supplementary Figures S6c5-8, spots of bone polish appear together with bright greasy polish along the same working edge.

3.2 Micro-residue results

The most common residues identified on our butchery tools were blood, fat, meat, tendons, bony fibers, soft tissues such as periosteum and, to a less extent, hair. The heterogeneous nature of residues produced while using experimental small tools in butchering reflected the different parts of the animal the flakes were in contact with during the process, as well as the type of actions and gestures performed with the small tools (cutting, scraping, slicing, etc.). The nature and distribution of the residues was also informative of the phase of the butchery process the small tools took part in (skinning, disarticulation, periosteum removal, etc.), due to the occurrence and combination of specific animal residues on the tool surfaces. As expected, the specific use-scars formed along the tool edges, as well as the prehension itself, actively influenced the distribution of butchery residues on the tool surfaces, along the edges of the flakes and in the prehensile areas.

Fat and soft collagen-rich fibers were abundantly noticed during the skinning phase together with fragments of hair. Fat distribution along the edge was invasive, and often formed a glossy-like film with linear smearing (Supplementary Figure S7a). Towards the prehensile areas fat often clustered with a fingerprint-like distribution (Supplementary Figure S7b). Fat droplets with a specific half-moon distribution were also been identified at high magnification (Supplementary Figure S7c).

When used in disarticulation activities, small flakes trapped blood and grease and, to a lesser extent, meat and ligament parts. Grease often covered significant parts of the tool due to the small dimension of the flakes as well as to the prehension applied. Meat, soft tissues (e.g. tendons or periosteum) and also hair and fat often clustered in patches along the edge but also far from it (Supplementary Figure S7d), close to the prehensile parts. In many cases, a mixture of fresh residues (fat, meat and soft collagen tissues) remained trapped as a compacted residue inside the very small edge-removal scars (Supplementary Figure S7e). Additionally, when the contact with bony soft tissues was more intense, for example in the case of disarticulation or removal of

fresh periosteum membrane, soft collagen fibers were mixed with fat and clustered in bands along the edge or in patches towards the prehensile areas. In some cases, bony soft tissues showed a transversal and/or longitudinal invasive distribution along the edge, in accordance with the gesture performed (Supplementary Figure S7f). It is important to note that the accidental contact with bone during the butchery often resulted in the presence of bony fibers trapped in the edge scars together with grease and meat.

3.3 Micro-FTIR and SEM-EDX results

After the butchery experiments, the micro-FTIR spectra of the replicas were analyzed under the same experimental conditions as archaeological artefacts.

Cryptocrystalline silica is the principal constituent of all the replicas and archaeological tools examined. All the reflectance spectra show a very intense peak at 1157 cm^{-1}, assigned to the Si-O stretching mode, and less intense bands at 798 and 469 cm^{-1}, attributed respectively to O-Si-O bending modes and to O-Si-O or O-Si-Al bending[93]. All peaks show an up-down reversal due to the reststrahlen effect[94,95].

The spectra of experimental tools after prolonged contact with flesh, meat and bone are reported next and show the range where absorption bands of residues were detected. Spectroscopic analysis was conducted at a number of locations on each of the experimental items. In the following figures, spectra suggesting the presence of micro residues of the worked material are compared to spectra of the same flint items without residues (black). The spectrum of a flint tool used for butchering is shown in Supplementary Figure S8a1.

Broad peaks at about 1640, 1513, 1445 cm^{-1}were detected. They are attributed to collagen, the most abundant protein of hide, and assigned respectively to C=O stretching (amide I), N-H bending and C-N stretching (amide II), C-H scissoring vibration of CH_2 and CH_3 groups. (IRUG database[88-91]). In addition, a very weak band was detected at about 1730 cm^{-1}, attributable to C=O stretching mode of peptide groups[96]. The amide III band, expected at around 1240 cm^{-1}, is not observed since it is overlapped by the very intense Si-O stretching of flint. The C-H stretching modes of the aforementioned molecules are present at 2913 and 2848 cm^{-1}. The weak shoulder at 877 cm^{-1} of the flint Si-O stretch suggests the existence of calcium carbonate ($CaCO_3$) traces whose CO stretching mode around 1450 cm^{-1} overlaps the C-H scissoring vibration of CH_2 and CH_3 groups.

The Supplementary Figure S8a2 shows the spectroscopic behavior of a flint replica that worked bone. The shoulder at the low frequency side of flint Si-O stretching mode (~ 913 cm^{-1}) suggests the presence of bone residue whose mineral component is hydroxyapatite of formula $Ca_5(PO_4)_3OH$[28,85].

Finally, Supplementary Figure S8a3 shows the micro-FTIR spectrum of a flint tool that worked meat, examined sometime after its use. The very weak broad band at approximately 1450 cm^{-1} indicates traces of $CaCO_3$ (see above), present also in the spectrum (black) without worked material residues. A doublet at 1575\1536 cm^{-1} is clearly observed and assigned to the C-O stretching mode of fatty acids (palmitate and or stearate) composing adipocere, the greasy lipid mixture into which animal fats are transformed[87].

A reference collection of EDX spectra was also created in order to identify the elemental composition of the analogous animal matters found on the archaeological assemblage (i.e., meat, hide, and bone). The methodological protocol included two kind of measurements: 1) in the first round we measured animal tissues mounted on aluminum stabs covered with carbon film to which the pure sampled substances were firmly attached; 2) in the second round, measures were taken directly on residues adhered to the flint surfaces of the items after the butchery experiments were performed.

This two-step analysis allowed elements characteristic of a single substance to be identified, which assisted us in the assignation and interpretation of the residues when they appear as superimposed and mixed amorphous masses of residues, as is the case after butchery activity. To verify the occurrence of a "fingerprint" combination of major chemical elements of each substance, we decided to take into consideration the chemical elements equal to or greater than 0.500 in weight %.

As shown in Supplementary Figure S8b and c, carbon (C) and oxygen (O) are the recurrent elements found in higher concentration on all the sampled residues, these two elements being present in all organic life. In addition to carbon and oxygen, meat is composed of about the same concentration of phosphorus (P) and sulphur (S), (Supplementary Figure S8b1). In hide, phosphorus appears in higher concentration, followed by calcium (Ca), potassium (K), sodium (Na) and sulphur (S) (Supplementary Figure S8b2). Bone, on the other hand, is represented by a well-established doublet of calcium-phosphorus in the fixed proportion of about 2:1[81,92,97](Supplementary Figure S8b3).

When measurements were taken on residues stuck on flint, the concentrations of elements varied slightly. An amorphous patch of flesh residues along the functional edge on a flake used to dismember and cut meat and hide showed concentrations of chlorine (Cl), silicon (Si), calcium (Ca), sulphur (S) and phosphorus (P) (Supplementary Figure S8c1). Accumulated fibers of hide on a flake used during the skinning process displayed concentrations of calcium (Ca), potassium (K), sulphur (S) and phosphorus (P) (Supplementary Figure S8c2). Scraping off the periosteum surrounding bones produced an accumulation of collagen fibers and bony tissues composed of calcium (Ca), phosphorus (P), and sulphur (S) (Supplementary Figure S8c3).

The results presented above highlight the complexity of precisely identifying the residues (e.g., distinguishing between meat and hide, notably on the archaeological material) using only the EDX analysis. In contrast to the clear correspondence between the Ca-P ratio and the bone micro-remains, animal tissues, including meat, hide and connective tissues, are characterized by a combination of elements in different proportions. Sulphur and phosphorus are ubiquitous, albeit in conjunction with other constituents[81,98-101], whose proportion may help to determine whether the residue is indeed animal as opposed to vegetal.

Two main factors affect the elemental composition of the animal tissues and the ratio between them. Concentrations of specific elements in animal tissues may vary among species or in accordance with geographical location, as the presence and the concentration of minerals in tissues are directly connected to the animal's diet.

Moreover, the presence or the absence of specific elements in animal tissues also depends on the amount of residue that is radiated and measured by the electron beam. The greater the amount of residue, the more intense the detected peaks will be.

In conclusion, SEM observations coupled with EDX measurements considerably improved our ability to identify and interpret ancient residues[101,102] but this combined technique shows its full potential when results are cross-correlated with data of other complementary approaches such as Optical Light Microscopy (OLM) and/or other chemical techniques.

Figures

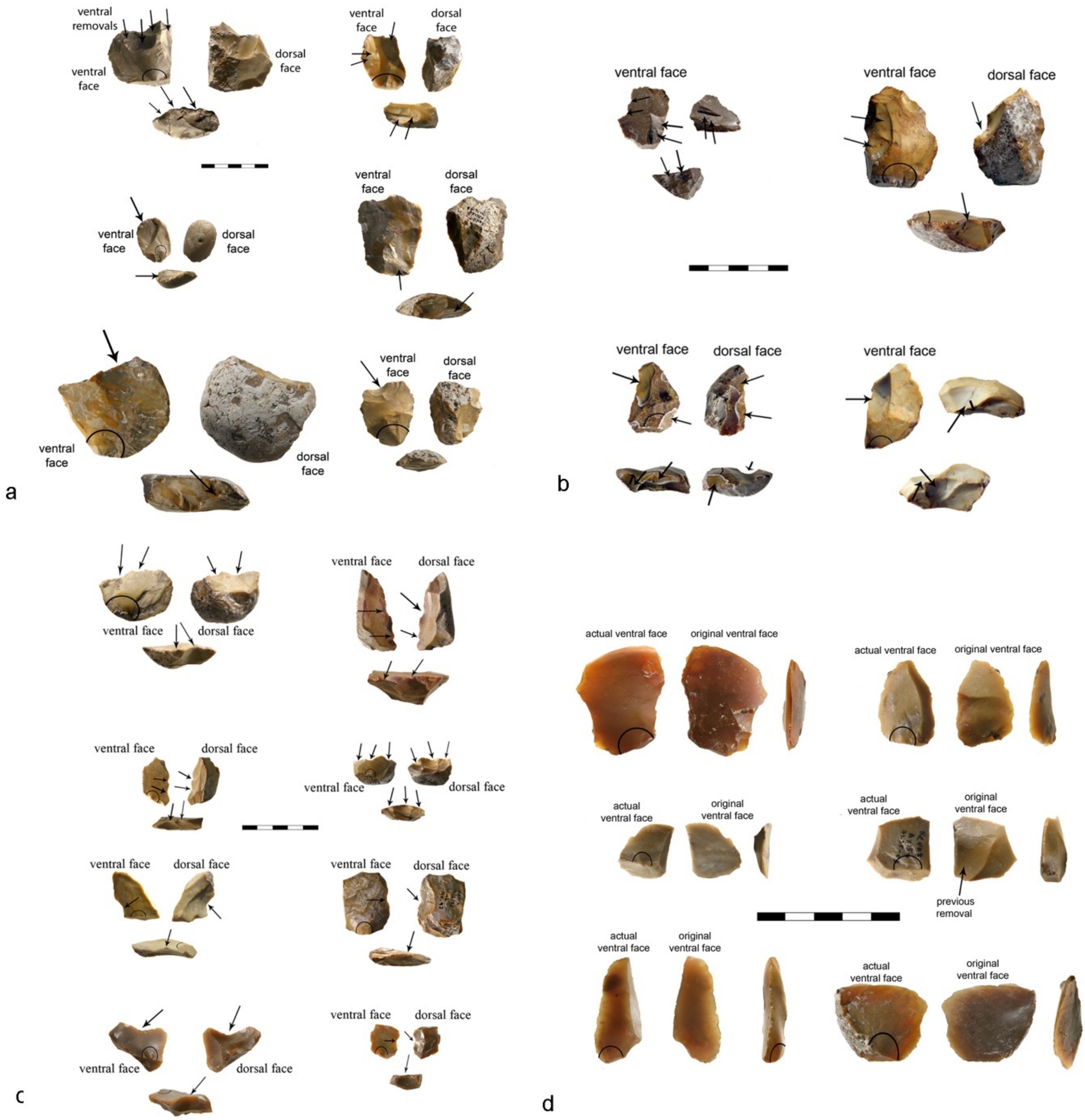

Supplementary Figure S1. Core-on-flakes (COF-FFs) and blanks produced from COFs. (**a**) COF-FFs with ventral removals. (**b**) COF-FFs with combined removals. (**c**) COF-FFs with dorsal removals. (**d**) Regular double ventral items and lateral double ventral items.

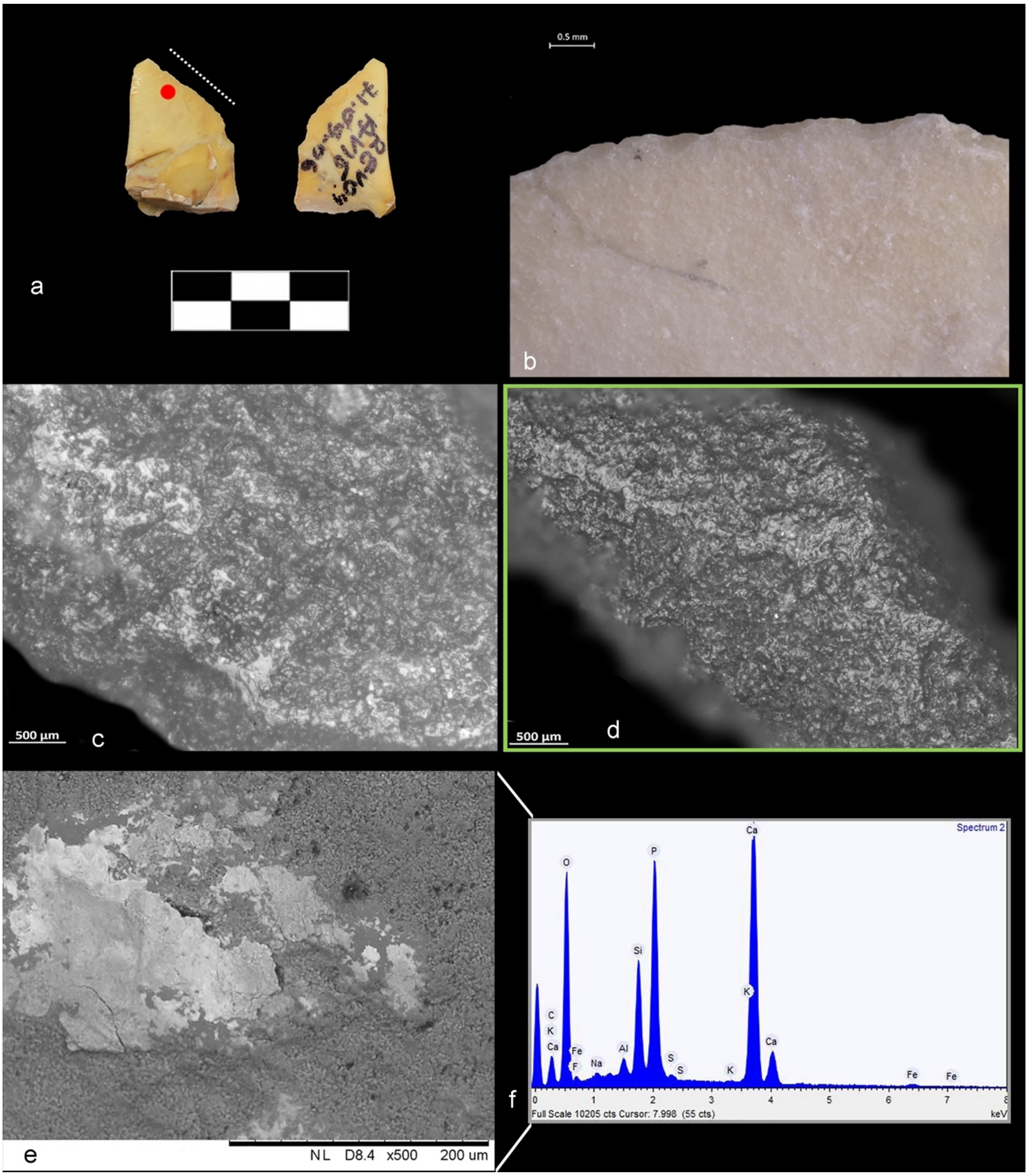

Supplementary Figure S2. **Use-wear traces and residues results on a small recycled flake.** (**a**) Double ventral Kombewa specimen Av15b 71.09-06 #51**.** (**b**) Edge damage. (**c**) Micro polish (magnification: 200X). (d) Micro polish after experimental activity of cleaning bone form meat (magnification: 200X). (**e-f**) SEM image of bone micro residue along the used edge and related EDX spectrum. Red dot show the EDX sampling point.

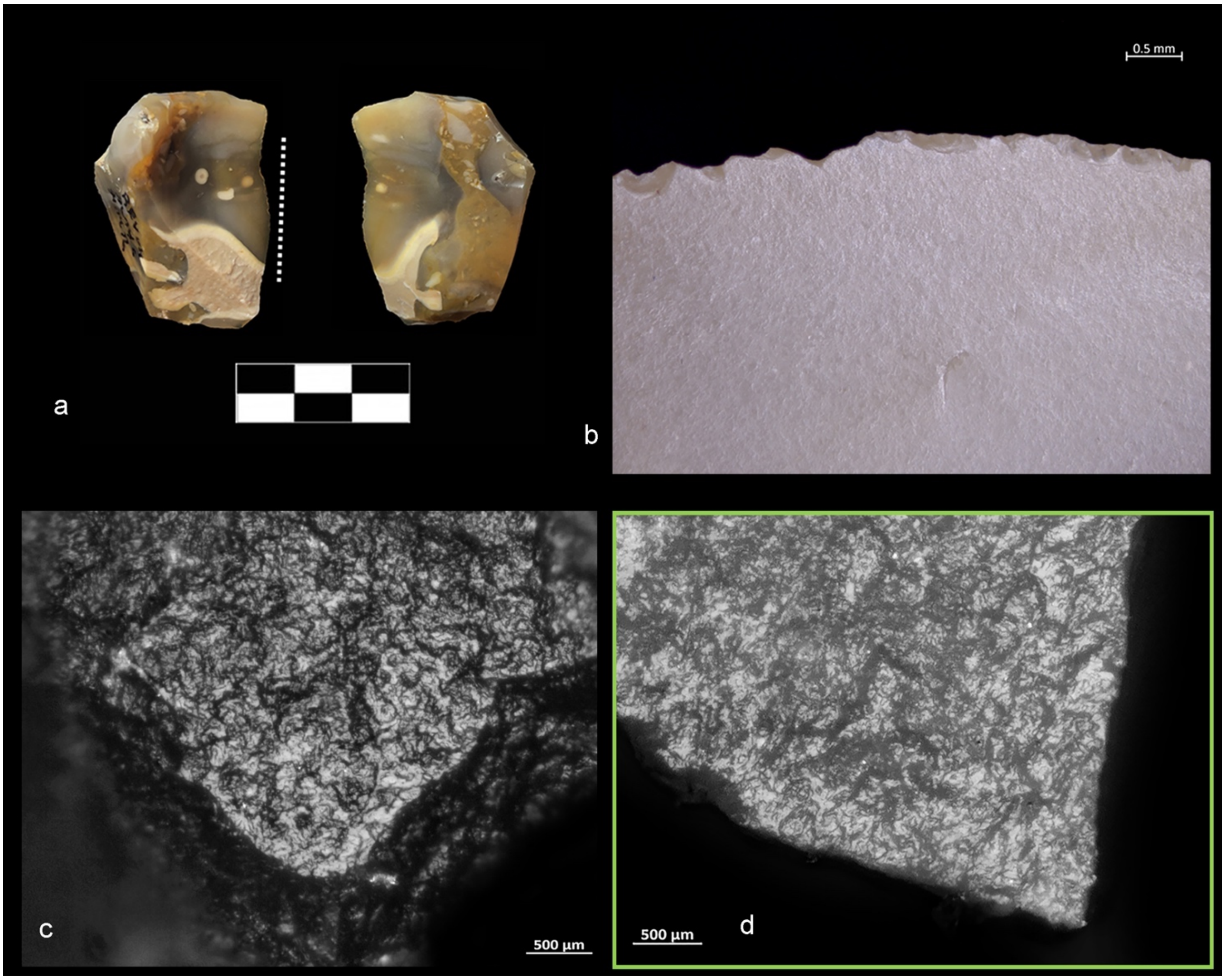

Supplementary Figure S3. Use-wear traces and residues results on a small recycled flake. (**a**) Double ventral Varia specimen AW14c 71.14-12 #70. (**a**) Edge damage. (**b**) Micro polish (magnification: 200X). (**c**) Micro polish after experimental activity of butchery (magnification: 200X).

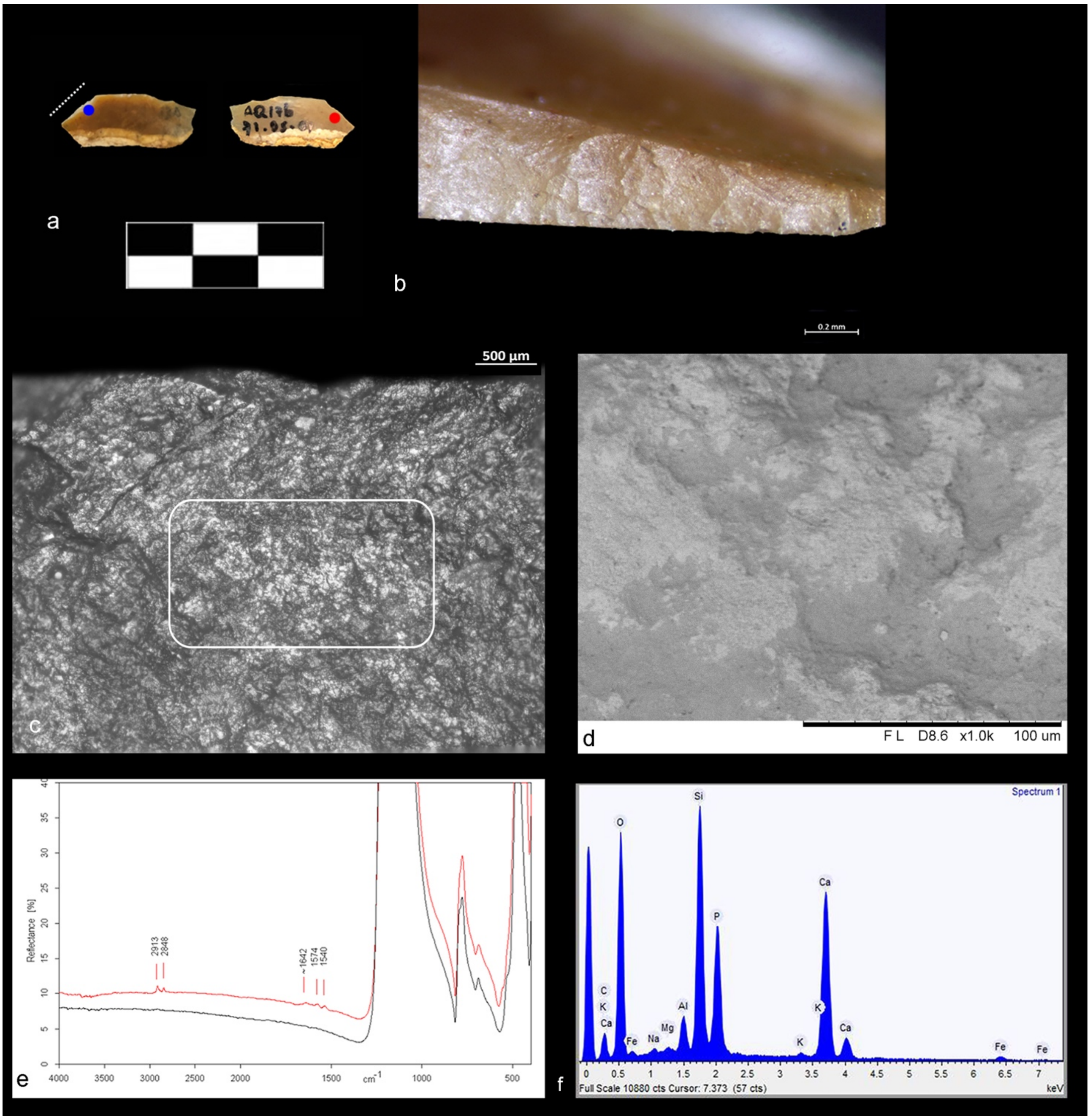

Supplementary Figure S4. Use-wear traces and residues results on a small recycled flake. (**a**) Double ventral regular specimen AQ17b 71.08-01 #32. (**b**) Edge damage located on a small rectilinear portion of the lateral side of the flake. (**c**) Micro polish located along the dorsal edge (magnification: 200X). (**d-e**) SEM image of bone micro residue along the used edge and related EDX spectrum. (**f**) Micro-FTIR spectrum (red) showing the absorption bands corresponding to the typical doublet absorption bands assigned to calcium salt carboxylate of saturated acids along with the C-H stretching of organic material and the band associated to Amide I. Black spectrum shows the fundamental mode of pure silica.
Red and blue dots show respectively the EDX and FTIR sampling points.

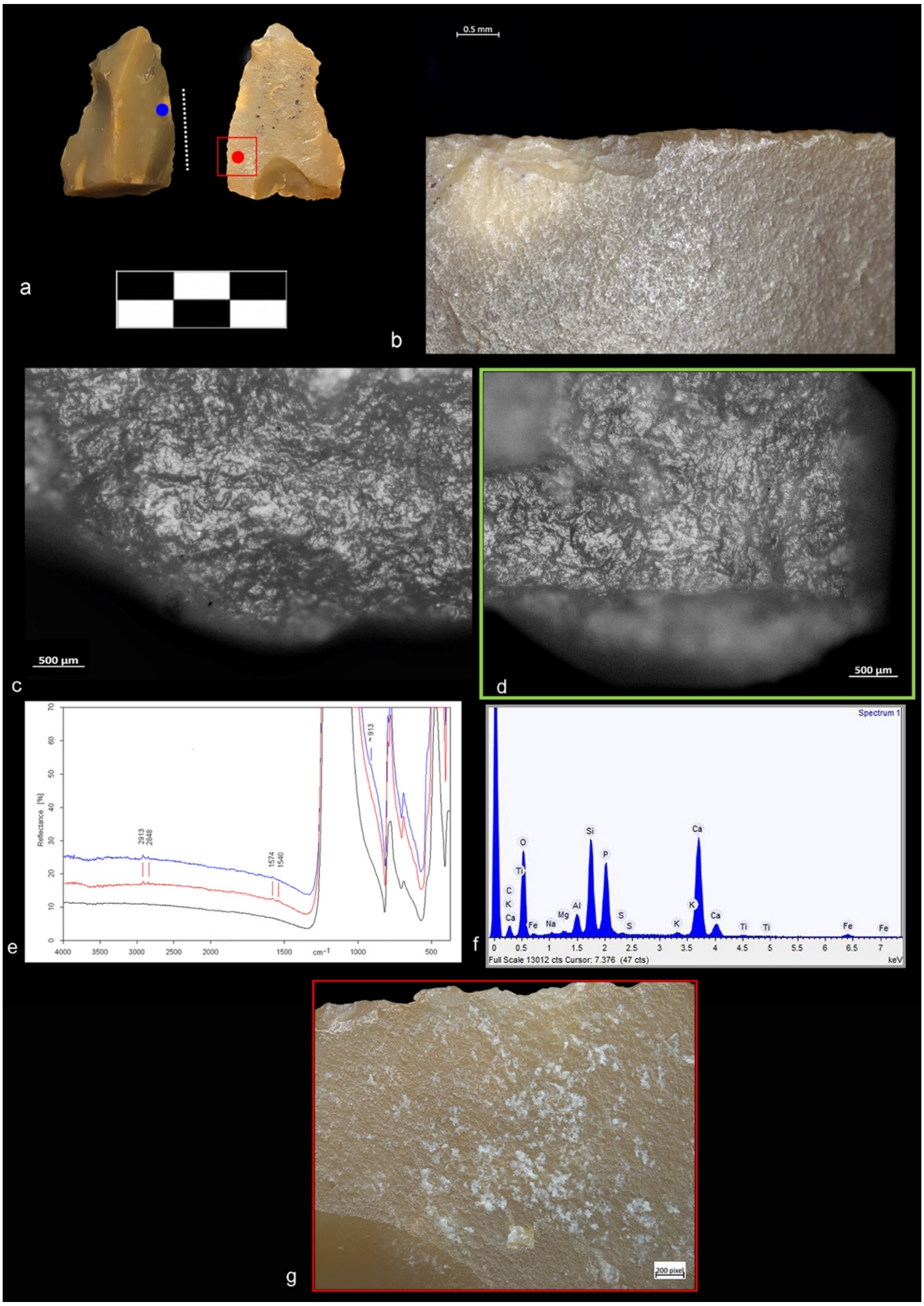

Supplementary Figure S5. **Use-wear traces and residues results on a small recycled flake.** (**a**) Double ventral Lateral specimen AS14a 71.11-08 #87. (**b**) Edge damage located on a small rectilinear portion of the lateral side of the flake. (**c**) Micro polish located along the dorsal edge (magnification: 200X). (**d**) Micro polish after experimental activity of removing periosteum (magnification: 200X). (**e**) Micro-FTIR showing the absorption bands corresponding to the typical doublet absorption bands assigned to calcium salt carboxylate of saturated acids along with the C-H stretching of organic material (red) and the shoulder assigned to bone (blue). Black spectrum shows the fundamental mode of pure silica. (**g-f**) Patch of compressed powder-like residue consistent with bony tissues (red square) and related EDX spectrum showing the bone composition. Red and blue dots show respectively the EDX and FTIR sampling points.

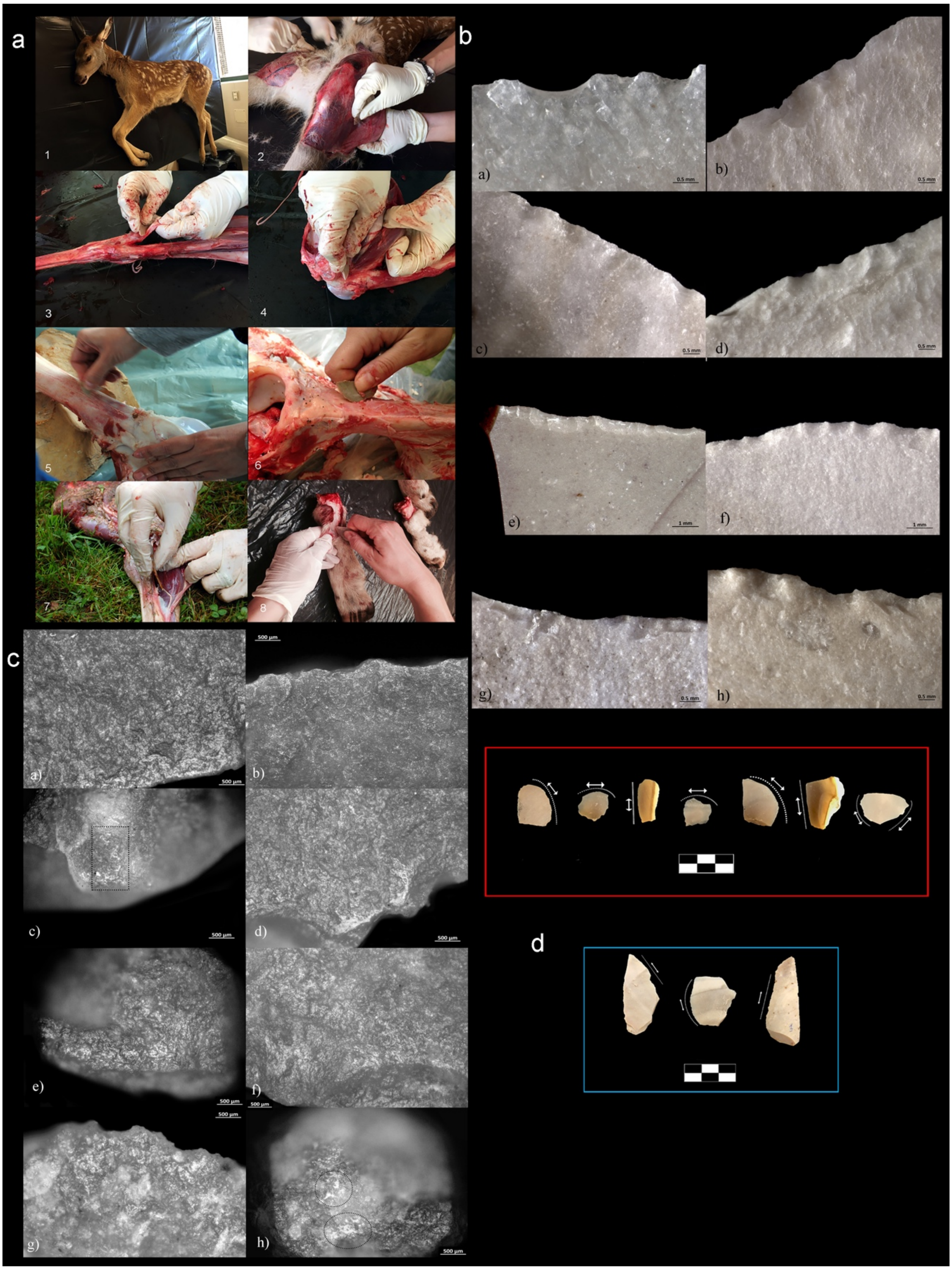

Supplementary Figure S6. Experimental activities, replicas and related use-wear traces. (**a**) Different moments in the processing of an animal carcass: **a**2) Skinning, **a**3) Cutting tendons, **a**4) Disarticulation, **a**5) Removing periosteum, **a**6) Scraping bone **a**7) Meat processing **a**8) Processing of a metapodial. (**b**1) Edge damage after skinning, (**b**2-4) Edge damage after butchery (**b**5) Edge damage after cleaning bone from meat (**b**6-8) Edge damage after removing periosteum (**c**1,2) Micro polish after skinning(magnification: 200X). (**c**3,4) Micro polish after butchery showing bone contact (magnification: 200X). (**c**5-8) Micro polish after removing periosteum (magnification: 200X). (**d**) Replicas used during the experimental activities. FV's reference collection (red square), replica used for this case study (blue square).

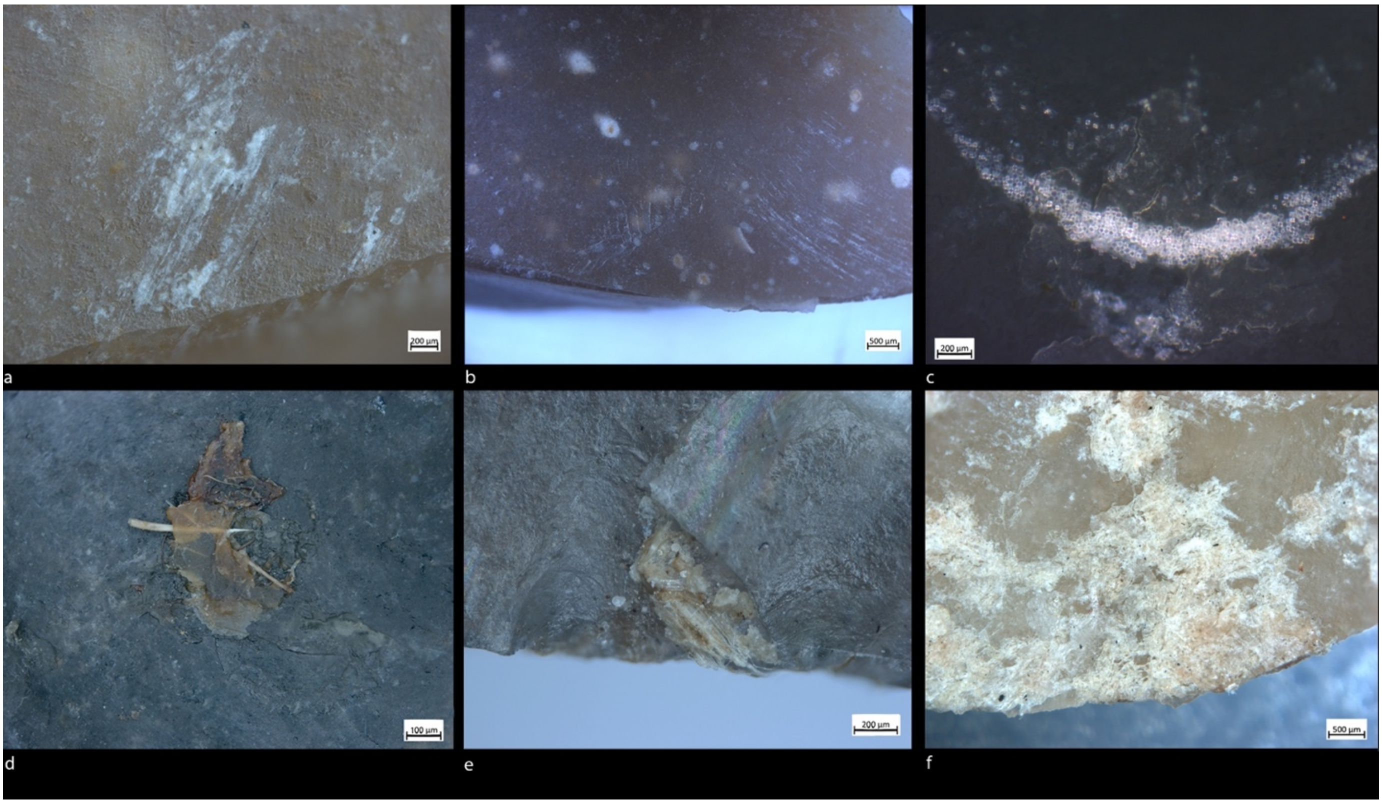

Supplementary Figure S7. Experimental residues from butchery activity on small tools. (**a**) Glossy film of whitish-beige fat residues from butchery with a linear distribution. (**b**) Grease with fingerprint distribution in a prehensile area. (**c**) Fat droplet distribution. (**d**) Grease, hair, meat and blood. (**e**) collagen fibers trapped under an edge scar. (**f**) Patches of whitish-beige collagen fibers on the edge and far from it.

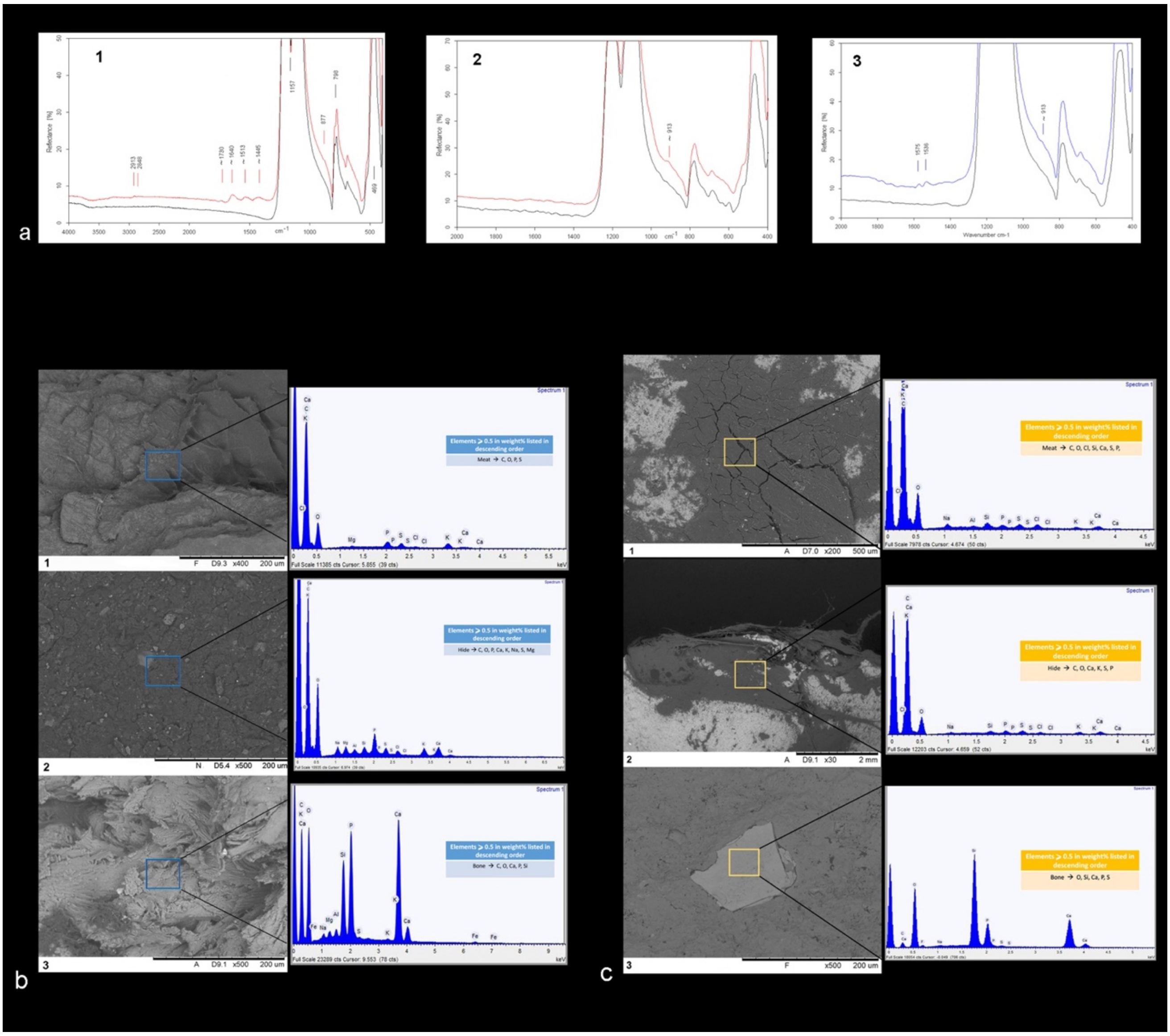

Supplementary Figure S8. **FTIR and EDX experimental reference collection**. (**a**) FTIR spectra of replica used for processing animal material: **a**1) butchery; **a**2) scraping bone; **a**3) bone cutting preserved under ash. (**b**) Pure animal substance and related EDX spectra: **b**1) meat; **b**2) hide; **b**3) bone. (**c**) residues adhered to the flint surfaces of the items after the butchery experiments and related EDX spectra: **c**1) meat; **c**2) hide; **c**3) bone.

Tables

	**Use-wear analysis**	**SEM-EDX analysis**	**FTIR analysis**
Number of analyzed items	283	53	53

Supplementary Table1: Number of analyzed items for each analytical method.

Worked Material	Cutting	Slicing	Scraping	Mixed	General Working	Indeterminable	Total
Fleshy tissues	1						**1**
Fleshy tissues+ bone	1		1				**2**
Bone			1				**1**
Connective tissues+ fresh hide	1						**1**
Soft material	48	1	2	1	1		**53**
Soft to medium material	14		5	1	2		**21**
Medium material	13		5	3			**22**
Medium to hard material	1		2				**3**
Indeterminable						5	**5**
Total							**107 (109FA)**

Supplementary Table 2. Interpreted function of small flakes.

Number	ID and Category	Use-wear interpretation	Residue description	Micro-FTIR spectra absorption bands (cm^{-1})	FTIR assignation	EDX elemental composition	EDX assignation
1	#2 Regular	Cutting soft material			/		/
2	#3 Regular	Cutting soft material	Patches of dense white deposit along the edge		/	Calcium and Phosphorous→ mineral part of bone (Hydroxyapatite)	Bone
3	#4 Regular	Scraping soft to medium material		~2913/2848→ν_{C-H} of organic material; ~913→ν_{P-O} of hydroxyapatite	C-H+Bone	Calcium and Phosphorous→ mineral part of bone (Hydroxyapatite)	Bone
4	#5 Regular	Cutting soft to medium material			/	Calcium and Phosphorous→ mineral part of bone (Hydroxyapatite)	Bone
5	#7 Regular	Cutting soft material			/		/
6	#8 Regular	Mixed on medium material	Dense white and yellow deposit along/far from the edge and pushed towards the prehensile area of the tool mixed with birefringent collagen fibers	~913→ν_{P-O} of hydroxyapatite	Bone	Calcium and Phosphorous→ mineral part of bone (Hydroxyapatite)	Bone
7	#10 Regular	Cutting soft material			/		/
8	#21 Regular	Cutting soft material	Dense and thick whitish deposit along and far from the edge associated to whitish/yellowish birefringent fibers.	~2913/2848→ν_{C-H} of organic material; ~1573/1537→ν_{C-O} of fatty acid calcium salt carboxylate	C-H+Adipocere	Calcium and Phosphorous→ mineral part of bone (Hydroxyapatite)	Bone
9	#22 Regular	Scraping medium material		~2913/2848→ν_{C-H} of organic material; ~913→ν_{P-O} of hydroxyapatite; ~1573/1537→ν_{C-O} of fatty acid calcium salt carboxylate; ~1645→$\nu_{C=O}$ of proteins (Amide I)	C-H+Bone+ Adipocere+ Amide I		/
10	#26 Regular	Cutting soft to medium material		~2913/2848→ν_{C-H} of organic material	C-H	Calcium and Phosphorous→ mineral part of bone (Hydroxyapatite)	Bone
11	#32 Regular	Scraping off animal fleshy tissues from bone during a butchery process		~2913/2848→ν_{C-H} of organic material; ~1573/1537→ν_{C-O} of fatty acid calcium salt carboxylate	C-H+Adipocere	Calcium and Phosphorous→ mineral part of bone (Hydroxyapatite)	Bone
12	#35 Regular	Cutting soft material		~2913/2848→ν_{C-H} of organic material; ~913→ν_{P-O} of hydroxyapatite; ~1573/1537→ν_{C-O} of fatty acid calcium salt carboxylate	C-H+Bone+ Adipocere	Calcium and Phosphorous→ mineral part of bone (Hydroxyapatite)	Bone
13	#37 Regular	Cutting soft material			/		/
14	#46 Regular	Cutting soft material		~2913/2848→ν_{C-H} of organic material; ~1573/1537→ν_{C-O} of fatty acid	C-H+Adipocere	Calcium and Phosphorous→ mineral part of bone (Hydroxyapatite)	Bone

				calcium salt carboxylate			
15	#51 Kombewa	Cutting off fleshy tissues from bone during a butchery process			/	Calcium and Phosphorous $\rightarrow$ mineral part of bone (Hydroxyapatite)	Bone
16	#52 Kombewa	Cutting soft to medium material		~2913/2848 $\rightarrow \nu_{C-H}$ of organic material; ~1573/1537 $\rightarrow \nu_{C-O}$ of fatty acid calcium salt carboxylate	C-H+Adipocere	Calcium and Phosphorous $\rightarrow$ mineral part of bone (Hydroxyapatite)	Bone
17	#53 Kombewa	Cutting soft material		~2913/2848 $\rightarrow \nu_{C-H}$ of organic material; ~913 $\rightarrow \nu_{P-O}$ of hydroxyapatite; ~1573/1537 $\rightarrow \nu_{C-O}$ of fatty acid calcium salt carboxylate	C-H+Bone+ Adipocere	Calcium and Phosphorous $\rightarrow$ mineral part of bone (Hydroxyapatite); Sulphur $\rightarrow$ Proteinaceous compounds (collagen)	Bone +soft animal tissues
18	#59 Kombewa	Cutting soft material	Patches of dense whitish deposit entrapped in edge-scars		/	Calcium and Phosphorous $\rightarrow$ mineral part of bone (Hydroxyapatite)	Bone
19	#60 Kombewa	Cutting soft to medium material			/		/
20	#61 Kombewa	Cutting medium material			/		/
21	#62 Kombewa	Cutting medium to hard material	Dense white deposit along and away from the edge as well as whitish/yellowish tissues smeared far from the edge towards the prehensile zone of the tool	~913 $\rightarrow \nu_{P-O}$ of hydroxyapatite	Bone	Calcium and Phosphorous $\rightarrow$ mineral part of bone (Hydroxyapatite)	Bone
22	#64 Overshot	Cutting medium material			/		/
23	#68 Overshot	Mixed on soft material		~2913/2848 $\rightarrow \nu_{C-H}$ of organic material; ~913 $\rightarrow \nu_{P-O}$ of hydroxyapatite; ~1573/1537 $\rightarrow \nu_{C-O}$ of fatty acid calcium salt carboxylate	C-H+Bone+ Adipocere	Calcium and Phosphorous $\rightarrow$ mineral part of bone (Hydroxyapatite)	Bone
23	#69 Varia	Mixed on medium material		~913 $\rightarrow \nu_{P-O}$ of hydroxyapatite	Bone		/
25	#70 Varia	Cutting animal fleshy tissues of medium consistency and contact with hide during a butchery process			/		/
26	#71 Varia	Cutting soft to medium material			/		/

27	#72 Varia	Cutting medium material	Dense and thick white deposit located far from the edge	~913→$\nu_{P\text{-}O}$ of hydroxyapatite	Bone	Calcium and Phosphorous→ mineral part of bone (Hydroxyapatite)	Bone
28	#73 Varia	Cutting soft to medium material		~2913/2848→$\nu_{C\text{-}H}$ of organic material; ~1573/1537→$\nu_{C\text{-}O}$ of fatty acid calcium salt carboxylate	CH+Adipocere	Calcium and Phosphorous→ mineral part of bone (Hydroxyapatite)	Bone
29	#79 Lateral	Mixed on soft to medium material	White tissues and birefringent fibers of collagen entrapped inside the edge damage	~913→$\nu_{P\text{-}O}$ of hydroxyapatite; ~1573/1537→$\nu_{C\text{-}O}$ of fatty acid calcium salt carboxylate	Bone+ Adipocere	Sulphur→ Proteinaceous compounds (collagen)	Collagen fiber
30	#80 Lateral	Cutting soft material		~2913/2848→$\nu_{C\text{-}H}$ of organic material; ~1573/1537→$\nu_{C\text{-}O}$ of fatty acid calcium salt carboxylate	C-H+Adipocere	Calcium and Phosphorous→ mineral part of bone (Hydroxyapatite)	Bone
31	#81 Lateral	Prolonged transversal contact with bone during a butchery process	Dense whitish deposit entrapped inside the edge damage and in a crack along the ridge of the dorsal side	~2913/2848→$\nu_{C\text{-}H}$ of organic material; ~913→$\nu_{P\text{-}O}$ of hydroxyapatite; ~1573/1537→$\nu_{C\text{-}O}$ of fatty acid calcium salt carboxylate	C-H+Bone+ Adipocere	Calcium and Phosphorous→ mineral part of bone (Hydroxyapatite)	Bone
32	#85 Lateral	Cutting soft to medium material		~2913/2848→$\nu_{C\text{-}H}$ of organic material; ~1573/1537→$\nu_{C\text{-}O}$ of fatty acid calcium salt carboxylate	C-H+Adipocere	Calcium and Phosphorous→ mineral part of bone (Hydroxyapatite)	Bone
33	#87 Lateral	Transversal contact with animal fleshy tissues and bone during a butchery process	Dense white deposit along the edge and inside use-scars as well as whitish/yellowish tissues smeared far from the edge towards the prehensile zones	~2913/2848→$\nu_{C\text{-}H}$ of organic material; ~913→$\nu_{P\text{-}O}$ of hydroxyapatite; ~1573/1537→$\nu_{C\text{-}O}$ of fatty acid calcium salt carboxylate	C-H+Bone+ Adipocere	Calcium and Phosphorous→ mineral part of bone (Hydroxyapatite)	Bone
34	#88 Lateral	Cutting medium material	Yellowish/reddish and whitish greasy amorphous animal structures, with birefringent collagen fibers entrapped in edge scars	~2913/2848→$\nu_{C\text{-}H}$ of organic material; ~913→$\nu_{P\text{-}O}$ of hydroxyapatite; ~1573/1537→$\nu_{C\text{-}O}$ of fatty acid calcium salt carboxylate	C-H+Bone+ Adipocere	Calcium and Phosphorous→ mineral part of bone (Hydroxyapatite)	Bone
35	#91 Lateral	Cutting soft to medium material			C-H+Adipocere	Calcium and Phosphorous→ mineral part of bone (Hydroxyapatite)	Bone
36	#95 Lateral	Cutting soft material		~2913/2848→$\nu_{C\text{-}H}$ of organic material; ~913→$\nu_{P\text{-}O}$ of hydroxyapatite; ~1573/1537→$\nu_{C\text{-}O}$ of fatty acid calcium salt carboxylate	C-H+Bone+ Adipocere		/

37	#97 Lateral	Cutting soft to medium material			C-H		/
38	#100 Lateral	Cutting medium material			C-H	Calcium and Phosphorous→mineral part of bone (Hydroxyapatite)	Bone
39	#102 Lateral	Cutting soft material	Whitish deposit along the edge pushed inside the use-scars	~2913/2848→ν_{C-H} of organic material; ~913→ν_{P-O} of hydroxyapatite; ~1573/1537→ν_{C-O} of fatty acid calcium salt carboxylate	C-H+Bone+ Adipocere	Calcium and Phosphorous→ mineral part of bone (Hydroxyapatite)	Bone
40	#105 Tabun snap	Cutting soft material		~2913/2848→ν_{C-H} of organic material; ~913→ν_{P-O} of hydroxyapatite; ~1573/1537→ν_{C-O} of fatty acid calcium salt carboxylate; ~1645→$\nu_{C=O}$ of proteins (Amide I);	C-H+Bone+ Adipocere+ Amide I		/
41	#109 Tabun snap	Cutting soft material		~2913/2848→ν_{C-H} of organic material; ~1573/1537→ν_{C-O} of fatty acid calcium salt carboxylate; ~1645→$\nu_{C=O}$ of proteins (Amide I);	C-H+Adipocere +Aimde I		/
42	#110 Tabun snap	Cutting soft to medium material		~2913/2848→ν_{C-H} of organic material; ~913→ν_{P-O} of hydroxyapatite; ~1573/1537→ν_{C-O} of fatty acid calcium salt carboxylate; ~1645→$\nu_{C=O}$ of proteins (Amide I);	C-H+Bone+ Adipocere+ Amide I	Calcium and Phosphorous→ mineral part of bone (Hydroxyapatite); Sulphur→ Proteinaceous compounds (collagen)	Bone +soft animal tissues
43	#112 Proximal end	Scraping medium material		~2913/2848→ν_{C-H} of organic material; ~913→ν_{P-O} of hydroxyapatite; ~1573/1537→ν_{C-O} of fatty acid calcium salt carboxylate; ~1645→$\nu_{C=O}$ of proteins (Amide I); 877→$\delta_{as}CO_3$ of calcite; ~1450→$\nu_{as}CO_3$ stretching of calcite	C-H+Bone+ Adipocere+ Amide I+ Calcium carbonate	Calcium and Phosphorous→ mineral part of bone (Hydroxyapatite); Sulphur→Proteinaceous compounds (collagen)	Bone + soft animal tissues

44	#113 Proximal end	Cutting soft material		~2913/2848→ν_{C-H} of organic material; ~913→ν_{P-O} of hydroxyapatite; ~1573/1537→ν_{C-O} of fatty acid calcium salt carboxylate; 877→$\delta_{as}CO_3$ of calcite; ~1450→$\nu_{as}CO_3$ stretching of calcite	C-H+Bone+ Adipocere+ Calcium carbonate	Calcium and Phosphorous→ mineral part of bone (Hydroxyapatite)	Bone
45	#115 Bulb shaving	Cutting soft to medium material		~2913/2848→ν_{C-H} of organic material; ~913→ν_{P-O} of hydroxyapatite; ~1573/1537→ν_{C-O} of fatty acid calcium salt carboxylate	C-H+Bone+ Adipocere	Sulphur→Proteinaceous compounds (collagen)	Soft animal tissues
46	#116 Bulb shaving	Cutting soft to medium material		~2913/2848→ν_{C-H} of organic material; ~913→ν_{P-O} of hydroxyapatite; ~1573/1537→ν_{C-O} of fatty acid calcium salt carboxylate; ~1645→$\nu_{C=O}$ of proteins (Amide I)	C-H+Bone+ Adipocere +Amide I		/
47	#118 Bulb shaving	Cutting soft material			C-H		/
48	#123 Reversed lateral	Cutting medium material		~2913/2848→ν_{C-H} of organic material; ~913→ν_{P-O} of hydroxyapatite; ~1645→$\nu_{C=O}$ of proteins (Amide I);	C-H+ Bone+Amide I		/
49	#126 Reversed lateral	Cutting soft material		~2913/2848→ν_{C-H} of organic material; ~913→ν_{P-O} of hydroxyapatite; ~1573/1537→ν_{C-O} of fatty acid calcium salt carboxylate	C-H+Bone+ Adipocere		/
50	#128 Reversed lateral	Scraping medium to hard material		~2913/2848→ν_{C-H} of organic material; ~913→ν_{P-O} of hydroxyapatite	C-H+Bone	Calcium and Phosphorous→ mineral part of bone (Hydroxyapatite)	Bone
51	#129 Reversed lateral	Cutting soft material		~2913/2848→ν_{C-H} of organic material; ~913→ν_{P-O} of hydroxyapatite; ~1573/1537→ν_{C-O} of fatty acid calcium salt carboxylate; ~1645→$\nu_{C=O}$ of proteins (Amide I)	C-H+Bone+ Adipocere+ Amide I	Calcium and Phosphorous→ mineral part of bone (Hydroxyapatite)	Bone

52	#131 Reversed lateral	Mixed on medium material		~2913/2848→ν_{C-H} of organic material; ~913→ν_{P-O} of hydroxyapatite; ~1573/1537→ν_{C-O} of fatty acid calcium salt carboxylate	C-H+Bone+ Adipocere		/
53	#132 Reversed lateral	Cutting medium material			/		/

Supplementary Table 3. List of small recycled flakes subjected to chemical analysis and related residue and use-wear assignation.
